# Up-regulation of HCN2 channels in a thalamocortical circuit mediates allodynia in mice

**DOI:** 10.1093/nsr/nwac275

**Published:** 2022-11-30

**Authors:** Jun-Ma Yu, Rui Hu, Yu Mao, Yingju Tai, Sen Qun, Zhi Zhang, Danyang Chen, Yan Jin

**Affiliations:** Department of Anesthesiology, The Third Affiliated Hospital of Anhui Medical University (The First People's Hospital of Hefei), Hefei 230061, China; Department of Anesthesiology, The Third Affiliated Hospital of Anhui Medical University (The First People's Hospital of Hefei), Hefei 230061, China; Department of Anesthesiology, The First Affiliated Hospital of Anhui Medical University, Hefei 230022, China; Department of Biophysics and Neurobiology, Division of Life Sciences and Medicine, University of Science and Technology of China, Hefei 230036, China; Department of Biophysics and Neurobiology, Division of Life Sciences and Medicine, University of Science and Technology of China, Hefei 230036, China; Stroke Center and Department of Neurology, The First Affiliated Hospital of USTC, Division of Life Sciences and Medicine, University of Science and Technology of China, Hefei 230036, China; Department of Biophysics and Neurobiology, Division of Life Sciences and Medicine, University of Science and Technology of China, Hefei 230036, China; Department of Biophysics and Neurobiology, Division of Life Sciences and Medicine, University of Science and Technology of China, Hefei 230036, China; Stroke Center and Department of Neurology, The First Affiliated Hospital of USTC, Division of Life Sciences and Medicine, University of Science and Technology of China, Hefei 230036, China

**Keywords:** chronic pain, HCN2 channels, neural circuit, *in vivo* recordings

## Abstract

Chronic pain is a significant problem that afflicts individuals and society, and for which the current clinical treatment is inadequate. In addition, the neural circuit and molecular mechanisms subserving chronic pain remain largely uncharacterized. Herein we identified enhanced activity of a glutamatergic neuronal circuit that encompasses projections from the ventral posterolateral nucleus (VPL^Glu^) to the glutamatergic neurons of the hindlimb primary somatosensory cortex (S1HL^Glu^), driving allodynia in mouse models of chronic pain. Optogenetic inhibition of this VPL^Glu^→S1HL^Glu^ circuit reversed allodynia, whereas the enhancement of its activity provoked hyperalgesia in control mice. In addition, we found that the expression and function of the HCN2 (hyperpolarization-activated cyclic nucleotide-gated channel 2) were increased in VPL^Glu^ neurons under conditions of chronic pain. Using *in vivo* calcium imaging, we demonstrated that downregulation of HCN2 channels in the VPL^Glu^ neurons abrogated the rise in S1HL^Glu^ neuronal activity while alleviating allodynia in mice with chronic pain. With these data, we propose that dysfunction in HCN2 channels in the VPL^Glu^→S1HL^Glu^ thalamocortical circuit and their upregulation occupy essential roles in the development of chronic pain.

## Teaser

The pain hypersensitivity is linked to functional connections between the thalamus and the cortical regions and we identified that the regulation of HCN2 channel encode the excitability of the VPL^Glu^→S1HL^Glu^ thalamocortical circuit which promotes the development of chronic pain.

## INTRODUCTION

Chronic pain is a pervasive, complex global health problem that is one of the principal causes of disease burden and disability [1]. Many analgesic drugs used in the treatment of chronic pain, including opioids and non-steroidal anti-inflammatory drugs (NSAIDs), are presently used for pain management during or after surgery [2]. However, side effects such as respiratory depression, nausea and vomiting, itching of the skin, and drug addiction due to long-term abuse continue to be problematic for both doctors and patients. Hence, it is essential to uncover innovative therapies for the treatment of chronic pain.

Nociceptive stimuli caused by tissue injury are transmitted to the central nervous system (CNS) via multiple pain-ascending pathways from the spinal cord [3]; and many brain regions such as the thalamus, primary somatosensory cortex (S1) [4,5], anterior cingulate cortex (ACC) [6–8], and insular cortex (IC) [9] are involved in the central regulation of chronic pain. Of these, the thalamus is the relay station for receiving information from multiple ascending pathways and transmitting nociceptive information to multiple cortices [10]. The ventral posterolateral nucleus (VPL) is known to be the principal somatosensory nucleus of the thalamus and the VPL processes and integrates pain information while distinguishing sensory and emotional dimensions [11]. Studies in animals have shown that inhibiting extracellular glutamate release in the VPL attenuates mechanical allodynia in a mouse model of spared nerve injury (SNI) [12]. In addition, clinical studies have also revealed that targeted excitation of the VPL nucleus by deep-brain stimulation can be used to treat a variety of pain syndromes [13]. The cortex receives information integrated by the thalamus for pain processing [8], and thalamocortical arrhythmias comprise one of the key pathological mechanisms governing chronic pain [14]. Use of functional magnetic resonance imaging (fMRI) found that enhanced connections between the VPL and somatosensory cortex in patients with chronic back pain [15], and the accurate inhibition of these functional connections produced a reduction in clinical pain by transcranial direct-current stimulation [16]. Collectively, these studies indicate that the VPL and its related thalamocortical circuit connections are associated with nociceptive processing. However, the molecular mechanisms subserving the VPL-related neuronal circuits in the pathogenesis of chronic pain are still largely undetermined.

Hyperpolarization-activated cyclic nucleotide-gated (HCN) channels are voltage-gated cation channels that are composed of four isoforms (HCN1–4) [17]. Since HCN channels were first reported to regulate the rhythm of myocardial sinoatrial nodal cells [18], HCN channels have subsequently been identified as widely expressed in the central and peripheral nervous systems; they appear to be critical to a series of physiological processes and pathological diseases—including pain [19], depression [20], epilepsy [21], and learning and memory [22]. Regarding the role of HCN channels in the occurrence and information transmission of pain [23], recent studies have revealed that mice with a genetic deletion of HCN2 channels showed the elimination of action potential firing in small dorsal root ganglion (DRG) neurons [24]. In addition, the expression of HCN2 channels in DRG neurons was enhanced in a mouse model of chronic pain [25]. These findings provide strong evidence that HCN2 channels may constitute an analgesic target in the treatment of chronic pain. Although HCN2 channels are widely expressed in the thalamus [17], the mechanism(s) by which they regulate the activity of thalamocortical circuits in chronic pain remains largely unelucidated.

In the present study, we demonstrated that the upregulation of HCN2 channels promoted augmented excitability of glutamatergic neurons of the VPL (VPL^Glu^) in mouse models of chronic pain and that this effect resulted in hyperactivity of the glutamatergic neurons of the hindlimb primary somatosensory cortex (S1HL^Glu^). Our findings thus suggest that upregulation of HCN2 channels is essential in the development of chronic pain, and that blocking their activity in VPL^Glu^ neurons comprises a feasible approach for the development of a novel class of analgesic drugs.

## RESULTS

### Enhanced VPL^Glu^ neuronal activity in mouse models of chronic pain

**Figure 1.**
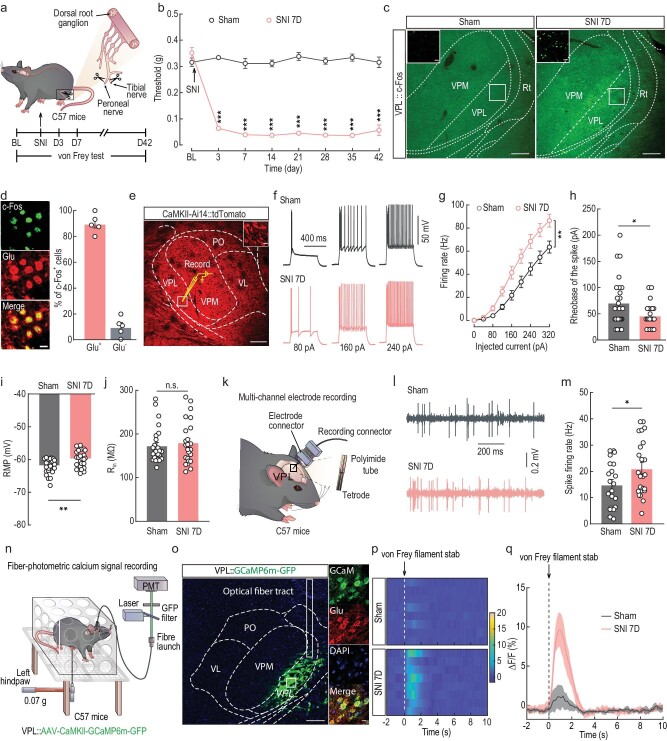
Increased excitability of VPL^Glu^ neurons in a mouse model of chronic neuropathic pain induced by SNI. (a) Schematic of the animal model of SNI. BL, baseline. (b) Time course of changes in response threshold to mechanical force via von Frey test in SNI mouse model of chronic pain. (c) Images showing the c-Fos expression in thalamic neurons in sham and SNI 7D mice. Scale bar, 200 μm. The insert depicts the area shown in the white box of the VPL. Scale bar, 20 μm. (d) Images (left) and statistics data (right) showing that c-Fos–positive neurons within VPL were mainly co-labeled with glutamate immunofluorescence. Scale bar, 10 μm. (e) Electrophysiological recordings from tdTomato-expressing VPL^Glu^ neurons in *CaMKII-Ai14* mice. Scale bar, 200 μm. The white box indicates the region shown in the box of the VPL. Scale bar, 10 μm. (f–h) Sample traces (f) and summarized data of firing rates (g), and rheobase of the spike (h) in VPL^Glu^ neurons recorded from sham and SNI 7D mice. (i and j) Summarized data of resting membrane potential (RMP, i) and input resistance (R_in_, j) in VPL^Glu^ neurons recorded from sham and SNI 7D mice. (k) Schematic of multi-channel electrode recordings in the VPL of freely moving mice. (l and m) Representative traces (l) and statistical data (m) showing the spontaneous spikes recorded from VPL^Glu^ neurons in sham and SNI 7D mice. (n) Schematic of the fiber photometry recordings. Ca^2+^ signal transients were recorded from GCaMP6m-expressing VPL^Glu^ neurons in C57 mice. (o) Representative images showing the injection site of AAV-CaMKII-GCaMP6m-GFP virus (left) and GCaMP6m-labeled neurons (green) co-localized with glutamate (Glu, red) immunofluorescence (right) within the VPL. Scale bars, 200 μm (left) or 10 μm (right). (p and q) Heatmaps (p) and the mean data (q) showing the change of VPL-Glu^GCaMP6m^ signals in sham and SNI 7D mice. The colored bar at the right in (q) indicates ΔF/F (%). All data are means ± SEM. *P < 0.05, **P < 0.01, ***P < 0.001. n.s., not significant. For detailed statistics information, see Table S1.

We established the spared nerve injury (SNI)-induced neuropathic pain model (Fig. 1a) and the complete Freund's adjuvant (CFA)-induced inflammatory pain model in mice (Fig. S1a), which are two well-known mouse models of chronic pain [26]. Seven days after SNI (SNI 7D) or three days after CFA injection (CFA 3D), the mice displayed a significant reduction in their mechanical threshold to allodynia by von Frey tests (Fig. 1b, Fig. S1b).

The VPL is classically described as a major relay station for pain-signal transmission that integrates pain-related information from regions such as the spinal cord and brainstem [27]. We therefore investigated neuronal activity in the VPL by observation of the expression of c-Fos, an immediate-early gene marker, and observed that c-Fos–positive neurons in the VPL were markedly elevated in SNI 7D mice and CFA 3D mice compared with control mice (Fig. 1c, Fig. S1c). Subsequent immunofluorescence staining showed that ∼90% of c-Fos signal was co-labeled with the glutamate antibody, which has been widely employed to identify cortical and subcortical glutamatergic neurons in other previous studies [28–30], in the VPL of both SNI 7D mice and CFA 3D mice (Fig. 1d, Fig. S1d). To validate the specificity of anti-Glu antibody, we injected a *VGluT2* promoter virus (AAV-VGLUT2-EGFP) and a *CaMKII* promoter virus (AAV-CaMKII-mCherry) into the VPL of C57 mice (Fig. S2a). After three weeks, immunofluorescence staining showed that mCherry-positive neurons were co-labeled with EGFP-positive neurons (Fig. S2b and c); the co-labeled neurons also co-localized with immunofluorescence signal of the glutamate antibody (Fig. S2d and e).

To examine the neuronal excitability of VPL^Glu^ neurons, we executed whole-cell patch-clamp electrophysiological recordings of red fluorescent protein tdTomato-expressing VPL^Glu^ neurons in brain slices from *CaMKII-Ai14* mice produced by *Ca^2+^/calmodulin-dependent protein kinase II*-*Cre* mice crossed with *Ai14* mice (Fig. 1e). We observed an increase in firing rate and a decrease in rheobase of current-evoked action potentials (tonic firing) (Fig. 1f–h), accompanied by a depolarized resting membrane potential (RMP) and no change in input resistance (R_in_) (Fig. 1i and j) in SNI 7D mice compared to that in sham mice. In addition, to further directly explore the role of VPL^Glu^ neuronal activity in freely moving mice, *in vivo* multi-channel electrode recordings were performed (Fig. 1k). We first identified the characteristics of spike waveforms by laser stimulation (473 nm, 20 Hz, 15 ms) of VPL^Glu^ neurons with VPL infusion of an adeno-associated virus (AAV) expressing Cre-dependent channelrhodopsin-2 (AAV-DIO-ChR2-mCherry) in the *CaMKII-Cre* mice (Fig. S3) under *in vivo* multi-channel optrode recordings, and we found that the spontaneous spike firing rate of VPL^Glu^ neurons was augmented in SNI 7D mice compared with that in sham mice (Fig. 1l and m). Moreover, to determine whether the activity of VPL^Glu^ neurons was sensitized to subthreshold stimuli, we measured Ca^2+^ signals in VPL^Glu^ neurons using the optical fiber photometer recordings of C57 mice harboring the virally expressed fluorescent Ca^2+^ indicator GCaMP6m (AAV-CaMKII-GCaMP6m) (Fig. 1n and o) and demonstrated that Ca^2+^ signals in VPL^Glu^ neurons increased rapidly following 0.07-g von Frey stimuli on the injured paws of SNI 7D mice (Fig. 1p and q). This suggested that VPL^Glu^ neurons became sensitized and activated more readily, which may have caused the increased spontaneous firing of VPL^Glu^ neurons during chronic pain.

Similarly, in CFA 3D mice, we also uncovered an elevation in current-evoked action potentials and a depolarized RMP with no change in R_in_ compared to saline mice (Fig. S4a–e). In addition, the enhanced activity of VPL^Glu^ neurons were also obtained in CFA 3D mice using *in vivo* multi-channel electrode and optical fiber photometry recordings (Fig. S4f–i). Collectively, these data suggested that VPL^Glu^ neuronal activity was enhanced under conditions of chronic pain.

### Manipulation of VPL^Glu^ neuronal activity regulates pain sensitization in mice

**Figure 2.**
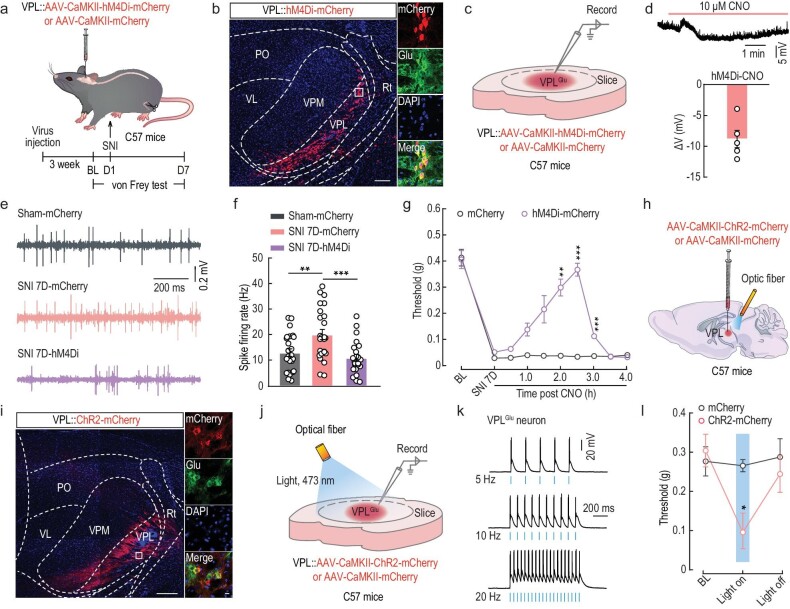
Manipulation of VPL^Glu^ neurons affects pain perception in mice. (a) Schematic of the experimental procedure. (b) Representative images of the injection site of AAV-CaMKII-hM4Di-mCherry (left) and mCherry-labeled neurons (red) co-labeled with glutamate immunofluorescence (green, right) within the VPL. Scale bars, 200 μm (left) or 20 μm (right). (c) Schematic of virus injection and the recording configuration. (d) Whole-cell recordings showing the effect of CNO on AAV-DIO-hM4Di-mCherry expressing VPL^Glu^ neurons. (e and f) Representative traces (e) and summarized data (f) of spontaneous spikes in VPL^Glu^ neurons of sham and SNI 7D mice infected with mCherry or hM4Di-mCherry within the VPL. (g) Effects of chemogenetic inhibition of VPL^Glu^ neurons on the pain threshold in SNI 7D mice. (h) Schematic of optogenetic experiments in C57 mice. (i) Images showing that the injection site within VPL of AAV-CaMKII-ChR2-mCherry (left) and mCherry-labeled neurons (red) co-localized with glutamate (Glu) immunofluorescence (right). Scale bars, 200 μm (left) or 10 μm (right). (j) Schematic of VPL injection of CaMKII-ChR2-mCherry in C57 mice and recording configuration in acute slices. (k) Sample traces of action potentials evoked by light (473 nm, 20 ms, blue line) recorded from VPL^Glu^ neurons in acute brain slices. (l) Effects of optogenetic activation of VPL^Glu^ neurons on the pain threshold in naive mice. All data are means ± SEM. *P < 0.05, **P < 0.01, ***P < 0.001. For detailed statistics information, see Table S1.

To further probe how VPL^Glu^ neurons participated in pain sensitization, we employed a chemogenetic inhibitory hM4Di virus (AAV-CaMKII-hM4Di-mCherry) under the regulation of a *CaMKII* promoter and the intraperitoneal injection of its ligand clozapine-N-oxide (CNO) to selectively inhibit VPL^Glu^ neurons in SNI 7D mice (Fig. 2a and b). Electrophysiological recordings from brain slices (Fig. 2c) revealed that the RMP of the hM4Di-mCherry^+^ VPL^Glu^ neurons was significantly hyperpolarized after perfusion of CNO (10 μM) (Fig. 2d), indicating that the chemogenetic virus was successfully expressed in VPL^Glu^ neurons. Under *in vivo* multi-channel electrode recordings, we also showed that chemogenetic inhibition of VPL^Glu^ neurons significantly reversed the SNI-induced enhancement of spontaneous firing (Fig. 2e and f), and prevented the SNI-induced allodynia that was manifested as a recovery of the mechanical pain threshold using von Frey tests (Fig. 2g) in SNI 7D mice. We additionally noted similar results in CFA 3D mice (Fig. S5).

We also infused an optogenetically stimulated, ChR2-expressing virus (AAV-CaMKII-ChR2-mCherry) into the VPL of C57 mice (Fig. 2h and i), and found that pulsed blue-light stimulations evoked action potentials in ChR2-mCherry^+^ VPL^Glu^ neurons (Fig. 2j and k) that led to a reduction in the threshold of mechanical pain in normal mice (Fig. 2l). These results confirmed the functional causality of the VPL^Glu^ neurons in mechanical allodynia of our mouse models of chronic pain.

### Upregulation of HCN2 channels enhances VPL^Glu^ neuronal activity in mouse models of chronic pain

**Figure 3.**
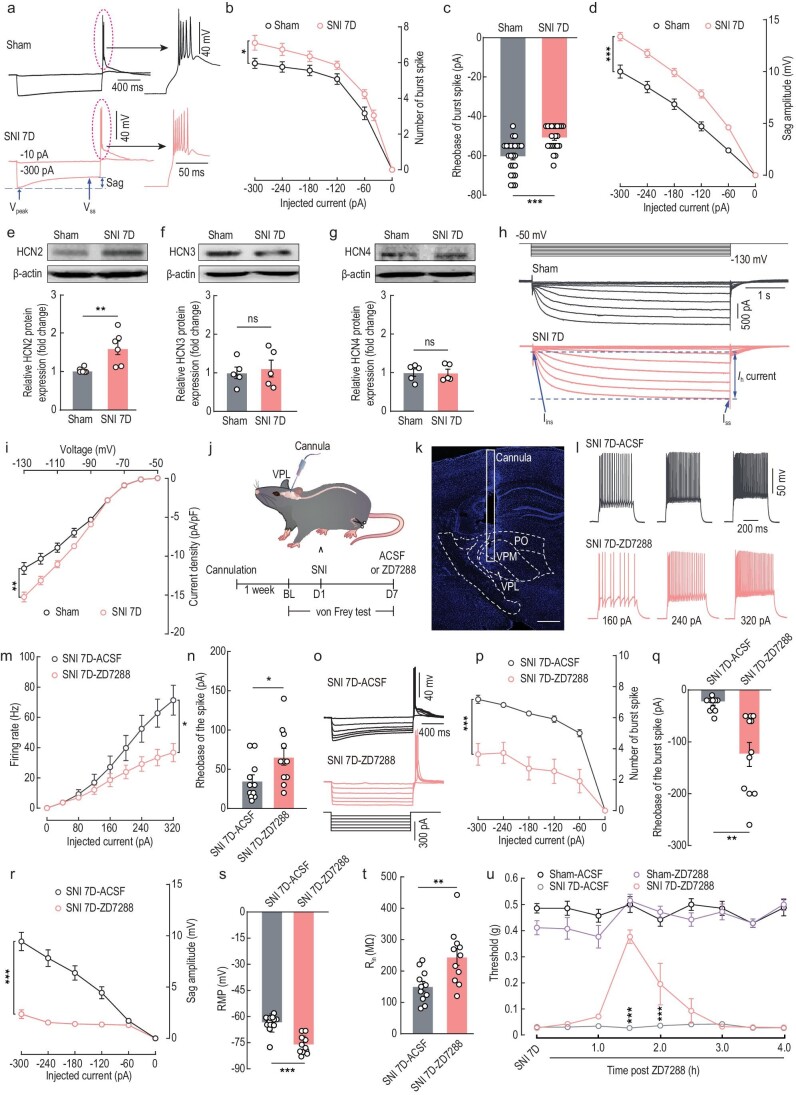
Inhibition of HCN2 channels attenuates VPL^Glu^ neuronal activity and relieves allodynia in SNI 7D mice. (a and b) Sample traces (a) and summarized data (b) of the spike in burst of VPL^Glu^ neurons recorded from *CaMKII-Ai14* sham and SNI 7D mice. ‘Sag’ was indicated at the end of the hyperpolarized potentials. (c) Statistical data of the rheobase of burst spike in VPL^Glu^ neurons recorded from sham and SNI 7D mice. (d) Summarized data of sag amplitude recorded from VPL^Glu^ neurons of sham and SNI 7D mice. (e–g) Western blotting of VPL lysates showing the HCN2 (e), HCN3 (f) and HCN4 (g) protein levels in sham and SNI 7D mice. (h) Representative traces of *I*_h_ currents recorded from VPL^Glu^ neurons in sham and SNI 7D mice. (i) Current density (pA*/*pF) of *I*_h_ is plotted against the voltage step. (j) Schematic of the experimental procedure. (k) Image showing the site of the cannula implanted in mice. Scale bar, 500 μm. (l and m) Sample traces (l) and summarized data (m) of firing rates recorded from VPL^Glu^ neurons in SNI 7D mice treated with ACSF or ZD7288. (n) Summarized data of the rheobase of the spike of VPL^Glu^ neurons in SNI 7D mice treated with ACSF or ZD7288. (o and p) Sample traces (o) and summarized data (p) of the spike in burst recorded from VPL^Glu^ neurons in SNI 7D mice treated with ACSF or ZD7288. (q–t) Statistical data of the rheobase of the burst spike (q), sag amplitude (r), RMP (s), and R_in_ (t) in VPL^Glu^ neurons from SNI 7D mice treated with ACSF or ZD7288. (u) Effects of pharmacological inhibition of HCN2 channels in the VPL on the pain threshold of sham and SNI 7D mice All data are means ± SEM. *P < 0.05, **P < 0.01, ***P < 0.001, n.s., not significant. For detailed statistics information, see Table S1.

We next investigated the molecular mechanisms underlying the elevation in VPL^Glu^ neuronal activity in mouse models of chronic pain. By delivering a series of hyperpolarized currents (from −10 pA to −300 pA, −10 pA/step, 500 ms) into VPL^Glu^ neurons (Fig. 3a), we found that the number of action potentials in the burst that appeared at the termination of pulses significantly increased, and that the rheobase of the burst firing diminished (Fig. 3b and c). Interestingly, the amplitude of a rebound depolarization (i.e., sag) at hyperpolarized potentials, measured as the difference between the peak membrane potential of the hyperpolarization (V_peak_) and the steady-state potential (V_ss_) near the end of the current steps, was larger in SNI 7D mice than in sham mice (Fig. 3d). The sag amplitude is a characteristic voltage change that indicates activation of the *I*_h_ current as mediated by HCN channels [31], and HCN channels are known to play a key role in controlling neuronal excitability and participate in pain sensitization [32].

There are four isoforms of HCN channels (HCN1–4) that are expressed in the CNS [31]. Using immunofluorescent staining, we found that HCN2, HCN3 and HCN4 were distributed in the VPL (Fig. S6). In addition, Western blots showed that protein levels of HCN2 in the VPL were upregulated in SNI 7D mice and CFA 3D mice (Fig. 3e, Fig. S7a), while no change in HCN4 protein level was observed in this group of mice, as compared to their respective controls (Fig. 3g, Fig. S7c). Moreover, no change in HCN3 protein levels was detected in SNI 7D mice compared to sham mice (Fig. 3f), and only a slight increase was detected in CFA 3D mice compared to saline mice (Fig. S7b). These results suggested that although HCN2, HCN3 and HCN4 channels are all distributed in the VPL, only HCN2 expression is significantly upregulated in both SNI and CFA mouse models.

Further immunofluorescent staining showed that HCN2 channels were highly expressed in VPL^Glu^ neurons in mice (Fig. S8a). To better understand the contribution of HCN2 channels in the VPL^Glu^ neurons to chronic pain, we then used electrophysiological recordings in whole-cell voltage-clamp mode as described previously [31] to specifically isolate HCN2 channel-mediated–*I*_h_ currents by holding membrane voltage at −50 mV with 5 s-long voltage steps from −50 mV to −130 mV (Fig. 3h), and these currents could be eliminated by bath application of the HCN channel-specific blocker ZD7288 (10 μM) (Fig. S8b and c). We found that the *I*_h_ current density was higher in the VPL^Glu^ neurons of SNI 7D mice compared to that of sham mice (Fig. 3i), indicating that HCN2 channel function was enhanced in SNI 7D mice. The function of HCN2 in cells is to enhance neuronal activity [33], and the enhanced *I*_h_ currents may, therefore, result in hyperactive tonic and burst firing of the VPL^Glu^ neurons in mice with chronic pain.

Next, we additionally examined whether blocking HCN2 channels exerted any effects on VPL^Glu^ neuronal activity and allodynia of SNI 7D mice (Fig. 3j and k). Using *in vitro* electrophysiological recordings in brain slices, we found that the number of current-evoked tonic spikes decreased while the rheobase of the spike increased (Fig. 3l–n); this was accompanied by decreased burst firings and sag amplitude, increased rheobase of burst spike, hyperpolarized RMP, and raised R_in_ (Fig. 3o–t) in VPL^Glu^ neurons of SNI 7D mice with ZD7288 microinjected intracranially into the VPL compared to SNI 7D mice with artificial cerebrospinal fluid (ACSF) treatment. In addition, behavioral tests showed the allodynia was relieved in SNI 7D mice after being treated with ZD7288 compared to those mice treated with ACSF (Fig. 3u). Similar results were observed in CFA 3D mice (Fig. S9). Taken together, these data suggested that the functional upregulation of the HCN2 channels may comprise one of the molecular mechanisms subserving allodynia in chronic pain conditions.

### Elucidation of an excitatory VPL^Glu^→S1HL^Glu^ pathway

**Figure 4.**
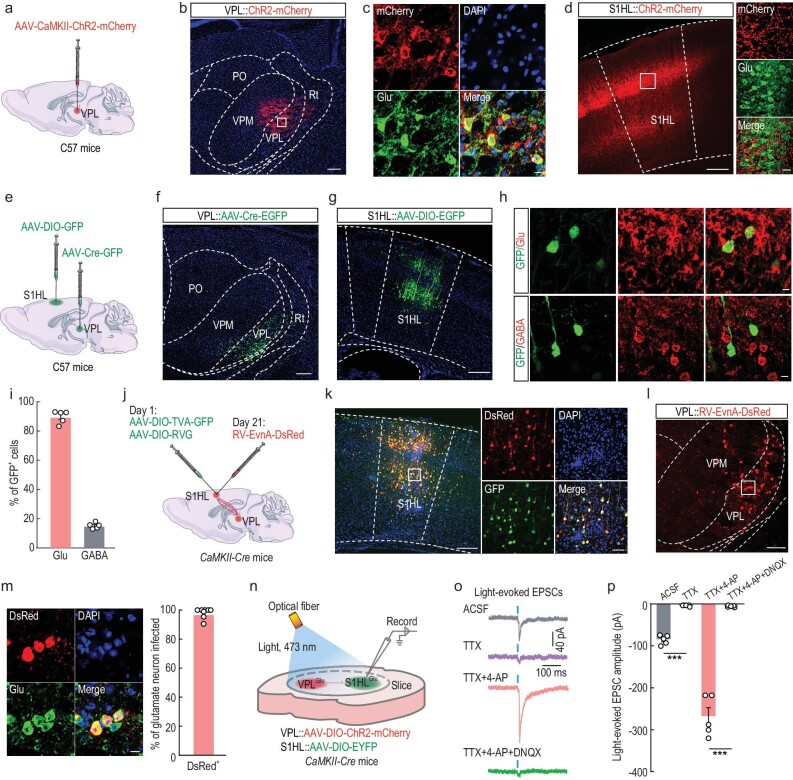
Dissection of the VPL^Glu^→S1HL^Glu^ circuit. (a) Schematic of viral injection. (b) Images showing that the injection site within the VPL by AAV-CaMKII-ChR2-mCherry. Scale bar, 200 μm. (c) Images showing ChR2-mCherry-labeled neurons (red) co-localized with glutamate immunofluorescence (Glu, green) within the VPL. Scale bar, 10 μm. (d) Representative images of mCherry^+^ fibers in the S1HL. Scale bars, 200 μm (left) or 20 μm (right). (e) Schematic of viral injection. (f and g) Representative images of viral expression within the VPL (f) and S1HL (g). Scale bars, 100 μm (f) or 200 μm (g). (h and i) Images (h) and statistical data showing that the majority of GFP-labeled neurons (green) within the S1HL are co-localized with glutamate immunofluorescence (Glu, red). Scale bar, 10 μm. (j) Schematic of the Cre-dependent retrograde trans-monosynaptic rabies virus tracing strategy in S1HL^Glu^ neurons of *CaMKII-Cre* mice. (k) Representative images of the injected site (left) and viral expression (right) within the S1HL. Starter cells (yellow) co-expressing AAV-DIO-TVA-GFP, AAV-DIO-RVG (green), and rabies RV-EnvA-ΔG-DsRed (red). Scale bars, 200 μm (left) and 60 μm (right). (l) Typical images showing that DsRed^+^ neurons (red) within VPL traced from the S1HL. Scale bar, 100 μm. (m) Images (left) and summarized data (right) showing that the DsRed^+^ neurons co-localized with glutamate immunofluorescence. Scale bar, 10 μm. (n) Schematic of virus injection and the recording configuration. (o and p) Representative traces (o) and summarized data (p) showing light-evoked EPSCs recorded from ipsilateral S1HL^Glu^ neurons held at −70 mV in the thalamocortical slices under the recording configuration shown in (n). All data are means ± SEM. ***P < 0.001. For detailed statistics information, see Table S1.

The most distinctive feature of the thalamus is its ability to transfer pain signals to the cerebral cortex for the integration and differentiation of pain [34]. To dissect the functional connection of this distinct pathway between the VPL and cortex, the AAV-CaMKII-ChR2-mCherry virus was infused into the VPL of C57 mice (Fig. 4a–c). Three weeks later, strong mCherry^+^ fibers were observed in the S1HL (Fig. 4d) that is known to mediate the sensory-discriminative aspects of hindlimb nociception [35], and not in the ACC, IC, or prefrontal cortex (PFC) (Fig. S10a and b). To confirm this VPL→S1HL projection, we injected anterograde monosynaptic AAV-Cre-GFP virus into the VPL and AAV-DIO-GFP virus into the S1HL of C57 mice (Fig. 4e and f). Three weeks later, we observed neurons with a positive green fluorescent protein (GFP^+^) in the S1HL (Fig. 4g) that was predominantly co-localized with the glutamate antibody (Fig. 4h and i).

To further scrutinize VPL→S1HL organization, we used a cell-type–specific retrograde trans-monosynaptic tracing system. Cre-dependent helper viruses (AAV-EF1α-DIO-TVA–GFP and AAV-EF1α-DIO–RVG) were injected into the S1HL of *CaMKII-Cre* mice (Fig. 4j, Fig. S10c), and after three weeks rabies virus (RV) (EnvA-pseudotyped RV-ΔG-DsRed) was injected into the same site (Fig. 4k); the presence of these helper viruses enabled the rabies virus to retrograde across monosynapses. We observed obvious DsRed-labeled neurons in the VPL, the posterior thalamic nucleus (PO), the zona incerta (ZI), contralateral S1HL, and the secondary somatosensory cortex (S2) (Fig. 4l, Fig. S10d and e); we noted that this signal was co-labeled with the glutamate antibody signal (Fig. 4m). Intriguingly, the DsRed-labeled neurons in the VPL were co-labeled with glutamate and HCN2 antibodies (Fig. S10f). These findings demonstrated a VPL^Glu^→S1HL^Glu^ thalamocortical pathway.

To characterize the functional connections of the VPL^Glu^→S1HL^Glu^ pathway, optogenetic experiments were performed by injection of AAV-DIO-ChR2-mCherry into the VPL and AAV-DIO-EYFP into the S1HL (Fig. 4n). Whole-cell recordings in brain slices showed that brief light stimulation of ChR2-containing VPL^Glu^ terminals in the S1HL reliably elicited excitatory postsynaptic currents (EPSCs) in the S1HL^Glu^ neurons. These EPSCs were blocked by bath application of the brain slices with tetrodotoxin (TTX, 1 μM), and the blockage was rescued by the addition of the potassium-channel blocker 4-aminopyridine (4-AP, 4 mM) to the TTX treatment. This rescue effect could then be eliminated by adding the AMPA receptor antagonist 6,7-dinitroquinoxaline-2,3(1H,4H)-dione (DNQX, 20 μM) to the TTX/4-AP co-treatment (Fig. 4o and p). These results demonstrated that S1HL^Glu^ neurons received functional monosynaptic excitatory glutamatergic projections from VPL^Glu^ neurons.

### Functional role of the VPL^Glu^→S1HL^Glu^ pathway in mouse models of chronic pain

**Figure 5.**
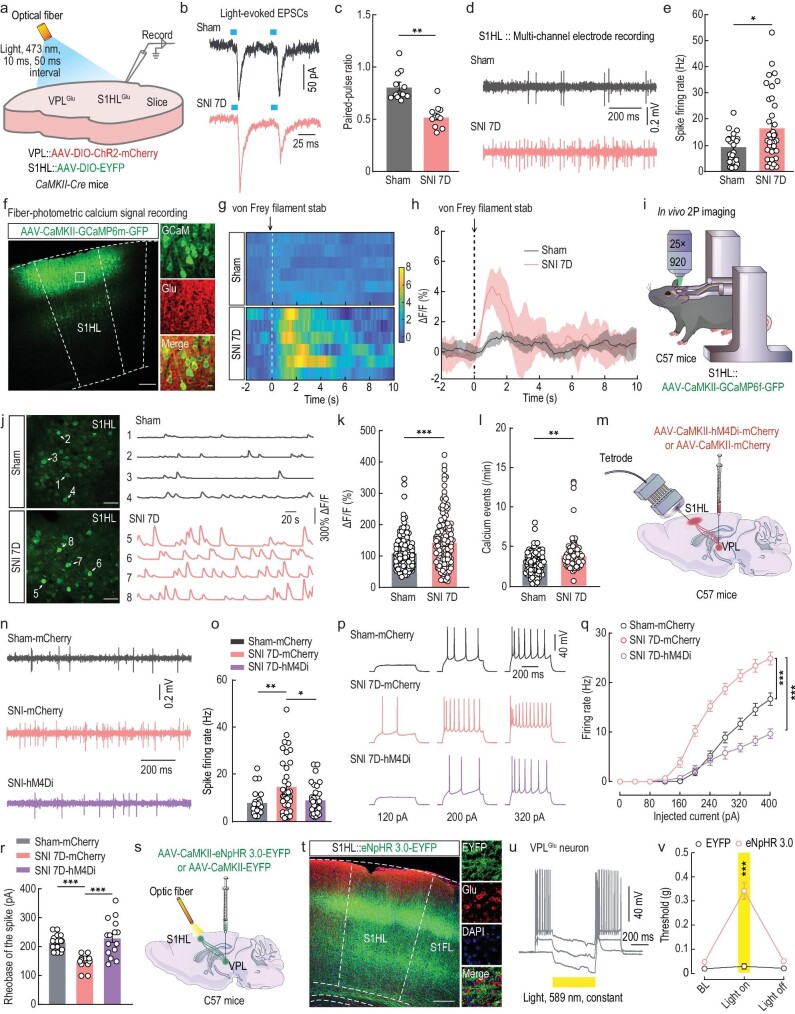
The VPL^Glu^→S1HL^Glu^ circuit modulates allodynia in SNI 7D mice. (a) Schematic of virus injection and the recording configuration. (b and c) Representative traces (b) and summarized data (c) of paired pulse ratios (PPRs) of light-evoked EPSCs in S1HL^Glu^ neurons. (d and e) Representative traces (d) and statistical data (e) showing the spontaneous spikes recorded from S1HL^Glu^ neurons in sham and SNI 7D mice. (f) Images showing that the injection site within the S1HL by AAV-CaMKII-GCaMP6m–(left) and GCaMP6m-labeled neurons (green) co-localized with glutamate (Glu, red) immunofluorescence within the S1HL (right). Scale bars, 200 μm (left) or 10 μm (right). (g and h) Heatmaps (g) and the mean data (h) showing the change of S1HL-Glu^GCaMP6m^ signals in sham and SNI 7D mice. The colored bar at the right in (g) indicates ΔF/F (%). (i) Schematic of the *in vivo* two-photon (2P) calcium image in head-restrained C57 mice with AAV-CaMKII-GCaMP6f-GFP expressing in S1HL^Glu^ neurons. (j) Representative images of 2P GCaMP6f^+^ imaging fields (left) and numbers matching spontaneous ΔF/F time series traces (right) from the imaging fields of S1HL^Glu^ neurons from sham and SNI 7D mice. Scale bar, 50 μm. (k and l) Statistical data showing the fluorescence intensity (k) and calcium influx events (l) of GCaMP6m-expressing S1HL^Glu^ neurons in sham and SNI 7D mice. (m) Schematic of virus injection and the recording configuration. (n and o) Representative traces (n) and summarized data (o) of spontaneous spikes in S1HL^Glu^ neurons of sham and SNI 7D mice infected with mCherry or hM4Di-mCherry within the VPL. (p and q) Sample traces (p) and summarized data (q) of current-evoked spikes recorded from S1HL^Glu^ neurons of sham and SNI 7D mice infected with mCherry or hM4Di-mCherry within the VPL. (r) Summarized data of the rheobase of the spike recorded from S1HL^Glu^ neurons of sham and SNI 7D mice. (s) Schematic of optogenetic experiments in C57 mice. (t) Left: images showing that the EYFP^+^ fibers within S1HL with VPL injection of AAV-CaMKII-eNpHR3.0-EYFP or AAV-CaMKII-EYFP. The background color (red) is the immunofluorescence staining of glutamate (Glu). Right: typical images showing the region in the white box in the S1HL. Scale bars, 200 μm (left) or 10 μm (right). (u) Sample traces of action potentials suppressed by light (589 nm, yellow line) recorded from VPL^Glu^ neurons in acute brain slices. (v) Effects of optogenetic inhibition of VPL^Glu^ neuronal fibers in the S1HL on pain thresholds. All data are means ± SEM. *P < 0.05, **P < 0.01, ***P < 0.001. For detailed statistics information, see Table S1.

To further detect whether the VPL^Glu^→S1HL^Glu^ pathway is overexcited under chronic pain conditions, we investigated the synaptic transmission of the VPL^Glu^→S1HL^Glu^ pathway through VPL injection with AAV-DIO-ChR2-mCherry and S1HL injection with AAV-DIO-EYFP in *CaMKII-Cre* mice (Fig. 5a). With a holding potential of −70 mV, we recorded the light-evoked EPSCs of EYFP^+^ S1HL^Glu^ neurons induced by photostimulating (473 nm, pulse width 10 ms, 50 ms interstimulus interval) ChR2^+^ VPL^Glu^ terminals in the S1HL in brain slices (Fig. 5b). The paired-pulse ratios of light-evoked EPSCs (PPR, ratio of EPSC2 over EPSC1) were decreased in S1HL^Glu^ neurons of SNI 7D compared to that in control mice (Fig. 5b and c). These results suggested increased presynaptic glutamate release from VPL^Glu^ neurons to S1HL^Glu^ neurons in the current models of chronic pain.

In light of our findings, we hypothesized that if S1HL neurons were innervated by VPL^Glu^ inputs in an excitatory state, then an increase in inputs under chronic-pain conditions should cause excitatory effects. To directly explore the role of S1HL^Glu^ neuronal activity in freely moving mice, *in vivo* multi-channel electrode recordings were executed in both chronic pain and control mice. We first identified the characteristics of spike waveforms by laser stimulation (473 nm, 20 Hz, 15 ms) of S1HL^Glu^ neurons with S1HL infusion of AAV-DIO-ChR2-mCherry virus in the *CaMKII-Cre* mice under *in vivo* multi-channel electrode recordings (Fig. S11), and found an increase in the firing rate of spontaneous spikes from S1HL^Glu^ neurons in SNI 7D mice compared with sham mice (Fig. 5d and e). Similar results were obtained in photometric calcium-signal recordings (Fig. 5f), which showed that the Ca^2+^ signals in S1HL^Glu^ neurons were increased after 0.07-g von Frey filament stimuli to the injured paws of SNI 7D mice (Fig. 5g and h, Video S1). To visualize Ca^2+^ signals in conscious mice, we injected the AAV-CaMKII-GCaMP6f-GFP virus into the S1HL (Fig. 5i), and *in vivo* two-photon calcium imaging showed that the fluorescence intensity and event of Ca^2+^ signals significantly increased in GCaMP6f-labeled S1HL^Glu^ neurons in SNI 7D mice (Fig. 5j–l). Analogous to the results with SNI 7D mice, we also observed decreased PPR of light-evoked EPSCs (Fig. S12a–c) and enhanced activity of S1HL^Glu^ neurons in CFA 3D mice (Fig. S12d–p). These results indicated that S1HL^Glu^ neuronal excitability was enhanced by increases in the excitatory inputs received from VPL^Glu^ neurons under conditions of chronic pain.

In order to evaluate the effects of manipulations of this neuronal circuit on S1HL^Glu^ neuronal activity and pain behaviors, we found that the enhanced activity of S1HL^Glu^ neurons was abolished by chemogenetic inhibition of VPL^Glu^ neurons, which was manifested as a recovery of c-Fos expression (Fig. S13a–c), the spontaneous firing of S1HL^Glu^ neurons using *in vivo* multi-channel electrode recordings and whole-cell recordings in brain slices in SNI 7D mice (Fig. 5m–r). In addition, the allodynia in SNI 7D mice was reversed by optical inhibition of VPL^Glu^ terminals in the S1HL following VPL injection of AAV-CaMKII-eNpHR-EYFP virus (Fig. 5s–v). Similar reversed effects were also observed in CFA 3D mice (Fig. S13d–j). Furthermore, we also found that optical activation of VPL^Glu^ fibers in the S1HL that expressed AAV-CaMKII-ChR2-mCherry virus induced allodynia in normal mice (Fig. S14). Taken together, our results revealed the functional causality in the VPL^Glu^**→**S1HL^Glu^ pathway in the development of chronic pain.

### Manipulation of HCN2 channels in VPL^Glu^ neurons affects pain behaviors

**Figure 6.**
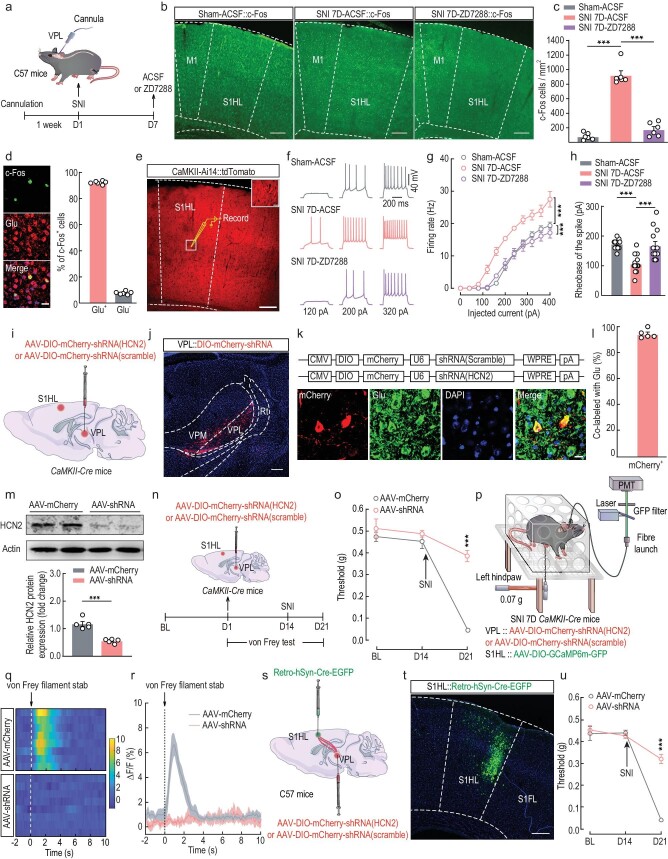
Functional knockdown of HCN2 channels in the VPL^Glu^→S1HL^Glu^ circuit rescues allodynia in SNI 7D mice. (a) Schematic of the experimental procedure. (b and c) Images (b) and statistical analysis (c) showing the distribution of c-Fos–positive neurons in the S1HL of sham and SNI 7D mice infused with ACSF or ZD7288 in the VPL. Scale bar, 200 μm. (d) Images (left) and statistical data (right) showing c-Fos–postive neurons (green) within S1HL were mainly co-labeled with glutamate immunofluorescence (Glu, red). Scale bar, 10 μm. (e) Electrophysiological recordings from tdTomato-expressing S1HL^Glu^ neurons in *CaMKII-Ai14* mice. Scale bar, 200 μm. The white box indicating the region shown in the box of the S1HL. Scale bar, 20 μm. (f and g) Sample traces (f) and summarized data (g) of current-evoked spike in S1HL^Glu^ neurons recorded from sham and SNI 7D mice treated with ACSF or ZD7288 infusion into the VPL. (h) Summarized data of the rheobase of current-evoked spike in S1HL^Glu^ neurons. (i) Schematic of viral injection. (j) Representative image showing the injection site within the VPL by AAV-DIO-mCherry-shRNA. Scale bar, 100 μm. (k) Top: schematic illustration of the construction strategy used for HCN2 channels downregulation with AAV-shRNA; Bottom: images showing mCherry-labeled neurons (red) co-localized with glutamate immunofluorescence (Glu, green) within the VPL. Scale bar, 10 μm. (l) Statistical data showing that mCherry-labeled neurons were mainly co-localized with glutamate immunofluorescence. (m) Western blotting of shRNA-scramble–infected and shRNA-HCN2–infected VPL lysates with antibodies against HCN2, β-actin. (n) Schematic of the experimental procedure. (o) Downregulation of HCN2 channels in VPL^Glu^ neurons alleviated allodynia of SNI 7D mice. (p) Schematic of the fiber photometry recordings in SNI 7D mice. (q and r) Heatmaps (q) and the mean data (r) showing the change of S1HL-Glu^GCaMP6m^ signals in SNI 7D mice infusion of AAV-DIO-mCherry-shRNA (HCN2) or AAV-DIO-mCherry-shRNA (scramble) with the VPL. The colored bar at the right in (q) indicates ΔF/F (%). (s) Schematic of virus injection in the VPL and S1HL. (t) Typical image showing the injection site within the S1HL. Scale bar, 200 μm. (u) Effects of downregulation of HCN2 channels in VPL^Glu^→S1HL^Glu^ pathway on pain thresholds in SNI 7D mice. All data are means ± SEM. ***P < 0.001. For detailed statistics information, see Table S1.

Based on our evidence that HCN2 channels were functionally enhanced in VPL^Glu^ neurons in mouse models of chronic pain, we hypothesized that the HCN2 channel was a potential analgesic target and investigated the role of HCN2 channels in regulating VPL^Glu^→S1HL^Glu^ circuit activity in pain perception. Immunohistochemistry (Fig. 6a–d) and whole-cell recordings (Fig. 6e–h) in brain slices showed that the respective increases in both c-Fos expression and neuronal activity were abrogated in S1HL^Glu^ neurons due to inhibition of HCN2 channels after intracranial microinjection of ZD7288 into the VPL of SNI 7D mice. To specifically block HCN2 channels in VPL^Glu^ neurons, we used an RNA interference (RNAi) viral vector (AAV-DIO-mCherry-shRNA (HCN2)) to knockdown VPL^Glu^ neuronal HCN2 protein levels and an AAV-DIO-mCherry-shRNA (scramble) as a control virus in *CaMKII-Cre* mice (Fig. 6i). Three weeks after the VPL injection of HCN2 RNAi virus (AAV-shRNA) (Fig. S15), immunofluorescence staining showed that ∼90% of mCherry^+^ neurons were co-localized with glutamate antibody in the VPL (Fig. 6j–l) and that VPL^Glu^ neuronal HCN2 protein level was reduced to ∼52.8% of that in the AAV control group (AAV-mCherry) (Fig. 6m).

Behavioral testing was performed to examine the blockade effects of HCN2 channels in chronic-pain mice, and we noted that, compared with the AAV-mCherry group, HCN2 knockdown markedly reversed the reduction in the mechanical pain threshold in SNI 7D mice (Fig. 6n and o). In addition, optical-fiber photometer recordings revealed that the Ca^2+^ signals remained unchanged following stimuli of a 0.07-g von Frey filament to the injured hindpaws of SNI 7D mice with HCN2 channel knockdown in the VPL (Fig. 6p–r, Video S2). To further corroborate the effect on pain sensitization by specific knockdown of the HCN2 channels in the VPL^Glu^ neurons projecting to the S1HL, we infused AAV-DIO-mCherry-shRNA (HCN2) virus into the VPL and retro-AAV-hSyn-Cre-EGFP into the S1HL (Fig. 6s and t) in C57 mice and found that the allodynia in these SNI 7D mice was still reversed (Fig. 6u). Similar results were obtained from CFA 3D mice (Fig. S16). Our collective findings from these disparate experimental manipulations suggested that the downregulation of HCN2 function in VPL^Glu^ neurons sufficiently and effectively rescued the enhancement of VPL^Glu^→S1HL^Glu^ circuit activity and allodynia in mouse models of chronic pain.

To further determine the effects of upregulation of HCN2 in the VPL→S1HL circuit on pain behaviors, we overexpressed the HCN2 protein in VPL^Glu^ neurons by injecting an AAV-DIO-HCN2-3xflag virus (AAV-HCN2) or an AAV-DIO-3xflag virus (AAV-control) into the VPL of *CaMKII-Cre* mice (Fig. S17a). After three weeks, we found that ∼90% of mCherry^+^ neurons co-localized with glutamate antibody in the VPL (Fig. S17b–d). Western blots of VPL tissues showed that HCN2 protein levels in the AAV-HCN2 group were increased to ∼159% of that in the AAV-control group (Fig. S17e). In addition, electrophysiological recordings showed that *I*_h_ current density and neuronal activity were increased in AAV-HCN2–expressing VPL^Glu^ neurons compared to those in VPL^Glu^ neurons expressing AAV-control (Fig. S17f–p). Post hoc immunostaining showed that the recorded neurons in the VPL labeled with neuronbiotin-488 in pipette solution were glutamatergic (Fig. S17q). Moreover, optical-fiber photometer recordings revealed that the Ca^2+^ signals of S1HL^Glu^ neurons were increased under HCN2 overexpression in VPL^Glu^ neurons, compared with AAV-control mice (Fig. S17r–t, Video S3).

Behavioral tests showed that the mechanical pain threshold was lower in mice with HCN2 overexpression in VPL^Glu^ neurons compared to that of AAV-control mice (Fig. S17u and v). Optogenetic inhibition of VPL^Glu^ terminal activity in the S1HL reversed pain sensitization in AAV-HCN2 mice with VPL infusion of AAV-DIO-eNpHR3.0-EYFP virus (Fig. S17w–y). These data suggested that upregulation of HCN2 function in the VPL^Glu^→S1HL^Glu^ pathway can generate pain behaviors.

### The cAMP signaling mediates HCN2 channel activity in VPL^Glu^ neurons in mouse models of chronic pain

HCN2 is regulated by membrane voltage and by intracellular cyclic adenosine monophosphate (cAMP) signaling. We exploited enzyme-linked immunosorbent assay of the cAMP and found that cAMP levels were significantly increased in the VPL of both SNI 7D and CFA 3D mice compared with that in the respective controls (Fig. S18a, Fig. S19a).

To understand the molecular mechanisms through which cAMP signaling may contribute to the HCN2-induced increase in VPL^Glu^ neuronal activity, the cAMP inhibitor, SQ22536, was intracranially microinfused into the VPL (Fig. S18b, Fig. S19b). Whole-cell recordings showed that *I*_h_ current density and neuronal activity were significantly decreased in VPL^Glu^ neurons of SQ22536-treated SNI 7D and CFA 3D mice compared with corresponding model mice treated with ACSF (Fig. S18c–m, Fig. S19c–m). Behavioral tests showed that SQ22536 application to the VPL significantly reduced mechanical pain thresholds in both SNI 7D and CFA 3D mice compared to model mice given ACSF (Fig. S18n, Fig. S19n). These results suggested that increased cAMP signaling can generate pain behaviors through HCN2 in mice with chronic pain.

## DISCUSSION

This study reveals that HCN2 channels perform an essential function in the molecular basis underlying chronic pain by regulating thalamocortical circuit activity. Central to this process is the upregulation of HCN2 channel activity that leads to increased excitability of VPL^Glu^ neurons; this is manifested as enhanced tonic and burst spikes, and results in the hyperactivity of S1HL^Glu^ neurons under chronic-pain conditions (Fig. S20).

The mechanistic understanding of molecular underpinnings and functional regulation of VPL^Glu^ neurons in pain remains largely elusive. Previous studies demonstrate that VPL neurons exhibit changes in rhythmic burst firing and increased excitability after spinal cord contusion injury [36], and that these activities are likely regulated by HCN-mediated *I*_h_ currents [37]. There are four HCN channel isoforms (HCN1–4) expressed in the CNS with distinct expression patterns [38,39]. The regulation of animal behaviors by HCN channels depends on channel subunits, subcellular localization, cell types, and brain regions. HCN2 channels have been reported to be widely expressed in the thalamus (including the VPL). Previous studies portray enhanced expressions of HCN2 channels in DRG neurons in mice with neuropathic and inflammatory pain [40,41]. Consistent with these studies, our findings reveal significant increases in HCN2 channel protein levels and in *I*_h_ currents of VPL^Glu^ neurons under chronic pain states. Blockade of HCN2 in the VPL decreases the activity of the VPL^Glu^→S1HL^Glu^ pathway and alleviates allodynia. These findings indicate that HCN2 channel-mediated neural circuit plasticity is a foundation for the development of chronic pain.

HCN2 channels are opened tonically at −65 mV, which leads to persistent inward and mixed Na^+^/K^+^ currents and contribute to generation of the tonic and burst firing of thalamic neurons [42,43]. In our study, we observed that inhibition of HCN2 channels reversed the elevation in tonic and burst firing of VPL^Glu^ neurons caused membrane hyperpolarization. In addition, HCN2 channels are known to be regulated by the cAMP signaling [44]. It has been reported that the intracellular cAMP was increased in somatosensory neurons in an animal model of painful diabetes [45]. In the current study, we found cAMP levels are increased in the VPL of SNI and CFA model mice, and that application of the cAMP blocker, SQ22536, decreases *I*_h_ currents and lowers the pain threshold in these mice. These results suggest that the cAMP signaling contributes to the effects of HCN2 channels on chronic pain.

The pain hypersensitivity is inextricably linked to functional connections between the thalamus and the cortical regions involved in nociceptive processing [35]. fMRI studies showed that functional connectivity in the thalamocortical network was enhanced in pain chronicity in humans [46]. The precise structure of thalamocortical circuits and their functional changes in chronic pain are poorly understood, and it remains unclear as to how ion channels in the thalamus that regulate thalamic neuronal firing affect the activity of the thalamocortical circuit. Herein, we identified an excitatory VPL^Glu^→S1HL^Glu^ thalamocortical neural circuit, the activity of which appears to be regulated by HCN2 channels. We also substantiated that downregulation of HCN2 channels can reverse the hyperactivity of the VPL^Glu^→S1HL^Glu^ neural circuit and relieve allodynia in SNI 7D and CFA 3D mice. Our findings thus delineate an ion channel-regulated neural circuit involved in the development of chronic pain.

Numerous brain regions are activated under chronic-pain conditions—such as PO, parafascicular nucleus, the paraventricular nucleus of the hypothalamus, and the lateral habenula [47–49]. With regard to the source of the enhanced activity of the VPL^Glu^→S1HL^Glu^ pathway in chronic-pain conditions, another theory entails the interaction between specific neurons within the different subnuclei of the thalamus or in other brain areas. Evidence from experimental animal studies revealed that optogenetically activated thalamic reticular nucleus (TRN) neurons projecting to the VPL reduced thermal hyperalgesia in chronic inflammatory pain in mice while reducing GABAergic transmission promoted pain [50]. Investigators have also recently shown that the optic stimulation of the NAc nucleus could alleviate the discharge of the VPL nucleus in the pain model of DRG neuropathic pain by reducing pain information traveling into the thalamus via the spinothalamic tract [51]. These results indicate that VPL neurons perform specific, essential roles for noxious-stimulation responses; the relationship between diverse brain regions and the VPL in various animal models of pain-sensing is a worthwhile investigative endeavor.

Different cortices receive projections from distinct subdivisions of the thalamus, which may perform various functions in the pathology of chronic pain [35]. In the current study, viral tracing showed that the VPL^Glu^ neurons mainly provided projections to the S1, but not the ACC, PFC, or IC; and the allodynia could be reversed by inhibition of VPL^Glu^ neuronal terminals in the S1HL. Although no projection from the VPL to ACC was observed in the current study, it should be noted that optical inhibition of ACC^Glu^ neurons is demonstrated to reduce the mechanical pain threshold in CFA mice [52]. A previous study has shown that the ACC receives inputs from the S1 and the activation of S1^Glu^ neuronal terminals in the ACC can increase the ACC^Glu^ neuronal response to noxious stimuli [53]. Hence, suppression of S1-projecting VPL^Glu^ neuronal activity could decrease the excitability of ACC^Glu^ neurons, thus alleviating pain behaviors.

In summary, our study illustrates the functional role of HCN2 channels in the regulation of the VPL^Glu^→S1HL^Glu^ circuit and provides novel insights into the neural circuit, cellular and molecular mechanisms underlying chronic pain. As the clinical analgesic drugs currently administered for chronic pain treatment remain unsatisfactory, our findings implicate HCN2 channels as potential therapeutic targets in the management of chronic pain.

## MATERIALS AND METHODS

For detailed materials and methods, please see the Supplementary data. All data were available in this manuscript.

### Animals

Male mice at 8 to 10 weeks of age were used in all experiments. All animal protocols were approved by the Animal Care and Use Committee of the University of Science and Technology of China (permit USTCACUC1901001).

### Animal models

SNI and CFA protocols were used to construct mouse models of chronic pain.

### Statistical analysis

Two-tailed Student's *t*-tests, one-way and two-way ANOVA with post hoc analyses were used to perform statistical comparisons. All data are presented as mean ± SEM.

## Supplementary Material

nwac275_Supplemental_Files
