# Supplementary material for: Up-regulation of HCN2 channels in a thalamocortical circuit mediates allodynia in mice
Source: Natl Sci Rev. 2022 Nov 30;10(2):nwac275. doi: 10.1093/nsr/nwac275 (PMC9945406; doi:10.1093/nsr/nwac275)
Supplement: nwac275_Supplemental_Files [file nwac275_supplemental_files.zip › Supplementary Data.docx]

**Supplementary Data for**

**Up-regulation of HCN2 channels in a thalamocortical circuit mediates allodynia in mice**

**Authors:** Jun-Ma Yu^1^†, Rui Hu^1^†, Yu Mao^2, 3^†, Yingju Tai^3^, Sen Qun^4^, Zhi Zhang^3^*, Danyang Chen^3^*, and Yan Jin^4^*

^1^Department of Anesthesiology, The Third Affiliated Hospital of Anhui Medical University (The First People’s Hospital of Hefei), Hefei 230061, China

^2^Department of Anesthesiology, The First Affiliated Hospital of Anhui Medical University, Hefei 230022, China

^3^Department of Biophysics and Neurobiology, Division of Life Sciences and Medicine, University of Science and Technology of China, Hefei 230036, China

^4^Stroke Center and Department of Neurology, The First Affiliated Hospital of USTC, Division of Life Sciences and Medicine, University of Science and Technology of China, Hefei 230036, China

†These authors contributed equally to this work.

**^*^Correspondence to:**

Zhi Zhang

E-mail: zhizhang@ustc.edu.cn

Danyang Chen

E-mail: danychen@ustc.edu.cn

Yan Jin

E-mail: [jinyan@ustc.edu.cn](mailto:jinyan@ustc.edu.cn)

The file includes:

Materials and Methods

Fig. S1 to S20

Video S1

Video S2

Video S3

Table S1

Table S2

**Materials and Methods**

**Animals.** All experiments were approved by the Care Committee of University of Science and Technology of China (USTC). C57BL/6J mice were obtained from Charles River; the ROSA26Ai14 Cre-dependent tdTomato reporter (Ai14) (1) and *CaMKII-ires-Cre* (2) were purchased from Jackson Laboratories. Mice aged 8-10 weeks were selected for all experiments. The mice were placed in a stable environment (temperature 23-25°C, humidity 50%), with five per cage. The mice were maintained under a 12-hour light-dark cycle (the lights on from 8:00 to 20:00) and were able to obtain water and food available ad libitum.

**Animal models of neuropathic pain.** All SNI mice were anesthetized with isoflurane. The skin and muscle of the left thigh were cut and separated to expose the sciatic nerve and its three terminal branches (sural nerve, common peroneal nerve and tibial nerve). The common peroneal nerve and common tibial nerve were ligated with nonabsorbent 4-0 chromium intestine. Then, the nerve was cut off, the cutting point was 2 mm from the ligation point, and the sural nerve was preserved. Finally, the wound was sutured and disinfected with iodophor. For the sham group, mice received the same operation but kept the nerve intact.

**Animal models of inflammatory pain.** CFA (20 µl) was injected into the plantar surface of the left hindpaws of mice under brief isoflurane anesthesia for inflammatory pain. Saline (0.9% NaCl) was injected as control.

**Behavioral testing.** Mechanical hyperalgesia was quantified by von Frey filament stimuli to the middle (for CFA mice) or the lateral edge (for SNI mice) of the plantar surface of the left hind paw. Individual mice were placed in a polymethyl methacrylate box (5 × 5 × 8 cm) on a wire grid floor. Mice were allowed to habituate to the testing environment for 1 h to achieve immobility before testing. A von Frey filament was inserted and the pressure was gradually increased. A nociceptive-like response was considered when paw withdrawal or licking was clearly observed. The mechanical pain threshold was calculated from the average of five applications.

**Virus and trace injection.** All virus injection procedures followed the Laboratory Biosafety Guidelines approved by the USTC. Before surgery, the mice were fixed in a stereotactic frame (RWD) under an intraperitoneal injection of pentobarbital (20 mg/kg). The core temperature of the mice was maintained at 36°C using a heating pad. A pulled glass microelectrode was backfilled with virus and connected to a 10-microliter syringe. The injection volume of different viruses varied from 100 to 300 nl depending on the viral titer and expression potential, and the infusion rate was 30 nl/min. The coordinates were included as medio-lateral (ML) from the midline (in mm), anterior--posterior (AP) from bregma and dorso-ventral (DV) from the pial surface of the brain. The relevant information of viruses is reported in Supplemental Table 2.

For monosynaptic anterograde tracing, rAAV-hSyn-EGFP-P2A-Cre-WPRE-pA(AAV-Cre-GFP, AAV2/1, 1 ×10^13^ vg/ml) was injected into the VPL (AP: −1.86 mm; ML: −0.86 mm; DV: −3.55 mm) of C57 mice to allow the virus to drive Cre-dependent transgene expression in the postsynaptic neurons. Simultaneously, rAAV-Ef1α-DIO-EGFP-WPRE-pA (AAV-DIO–GFP, AAV2/9, 5.08 × 10^12^ vg/ml) was injected into the S1HL (AP: −1.60 mm; ML: −0.35 mm; DV: −0.80 mm). After 3 weeks, mice that had been anesthetized with an intraperitoneal injection of pentobarbital (20 mg per kg) were transcardially perfused, and brain slices were prepared for tracing the GFP signal or co-staining with glutamate-specific antibodies or GABA-specific antibodies in the S1HL.

For retrograde monosynaptic tracing, a 200 nl volume of helper viruses containing rAAV-Ef1α-DIO-RVG-WPRE-pA (AAV-DIO–RVG, AAV2/9, 4.59 × 10^12^ vg/ml) and rAAV-EF1α-DIO-H2B-EGFP-T2A-TVA-WPRE-hGH-pA (AAV-DIO-EGFP, AAV2/9, 5.56 × 10^12^ vg/ml; 1:2) was injected into the S1HL of *CaMKII-Cre* mice. Three weeks later, 300 nl RV-EnvA-ΔG-DsRed (2 × 108 IFU ml^−1^) was injected into the S1HL at the same site. Seven days after the last injection, mice that had been anesthetized with pentobarbital were transcardially perfused, and brain slices (40 µm) were prepared for tracing the DsRed signal or costaining with glutamate-specific antibodies.

For chemogenetic manipulation, the rAAV-Ef1α-CaMKIIα-hM4D(Gi)-mCherry-WPRE-pA (AAV-CaMKII-hM4Di-mCherry, AAV2/9, 3.69 × 10^13^ vg/ml) virus was injected into VPL of C57 mice. Three weeks after viral injection, an intraperitoneal injection of CNO (5 mg/kg, Sigma) was given 30 min before the behavioral tests. rAAV-Ef1α-CaMKII-mCherry-WPRE-pA (AAV-CaMKIIα-mCherry, AAV2/9, 8.93 × 10^12^ vg/ml) was used as the control.

For optogenetic manipulation, rAAV-Ef1α-CaMKIIα-eNpHR3.0-EYFP-WPRE-pA (AAV-CaMKIIα-eNpHR-EYFP, AAV2/9, 1.18×10^13^ vg/ml) was injected into the VPL of C57 mice for optogenetic inhibition. In some experiments, rAAV-Ef1α-CaMKII-hChR2(H134R)-mCherry-WPRE-pA (AAV-CaMKII–ChR2–mCherry, AAV2/9, 1.63 × 10^13^ vg/ml, 200 nl) was used for optogenetic activation. For validation of the anti-Glu antibody specificity, rAAV-VGLUT2-EGFP-WPRE-hGH-pA (AAV-VGLUT2-EGFP, AAV2/9, 2.82×10^12^ vg/ml, 200 nl) and rAAV-Ef1α-CaMKII-mCherry-WPRE-pA (AAV-CaMKIIα-mCherry, AAV2/9, 8.93 × 10^12^ vg/ml, 200 nl) were injected into the VPL.

To knock down of HCN2 channels in VPL^Glu^ neurons, rAAV-CMV-DIO-(mCherry-U6)-shRNA (HCN2)-WPRE-pA (AAV-DIO-mCherry-shRNA (HCN2)), AAV2/9, 5.35×10^12^ vg/ml) was injected into the VPL of *CaMKII-Cre* mice. rAAV-CMV-DIO-(mCherry-U6)-shRNA(scramble)-WPRE-pA (AAV-DIO-mCherry-shRNA(scramble)), AAV2/9, 5.03×10^12^ vg/ml) was used as a control. To overexpress HCN2 channels in VPL^Glu^ neurons, rAAV-CMV-DIO-HCN2-3xflag-WPRE-PA (AAV-DIO-HCN2-3xflag, AAV2/9, 5.0 ×10^12^ vg/ml) was injected into the VPL of *CaMKII-Cre* mice, with rAAV-CMV-DIO-3xflag-WPRE-PA (AAV-DIO-3xflag, AAV2/9, 2.0×10^12^ vg/ml) infection used as a control. To manipulate HCN2 channels in the VPL^Glu^→S1HL^Glu^ pathway, rAAV-hSyn-CRE-EGFP-WPRE-hGH-pA (AAV2/R, 5.45×10^12^ vg/ml) was injected into S1HL to allow the virus to spread retrogradely to the upstream soma to express Cre and AAV-DIO-mCherry-shRNA (HCN2) was injected into VPL of C57 mice. Unless otherwise stated, all viruses were purchased from BrainVTA. All mice were transcardially perfused with 0.9% saline followed by ice-cold phosphate buffer (0.1 M) that contained 4% paraformaldehyde. Images of the signal expression were acquired with a confocal microscope (LSM 880, ZEISS). Animals with missed injections were excluded.

***In vivo* pharmacological approach.** After anesthesia by an intraperitoneal injection of pentobarbital (20 mg per kg), a cannula (diameter of 0.25 mm, length of 4.5 mm, RWD) was implanted into the VPL of C57 mice immobilized in a stereotaxic apparatus. The implant was secured to the skull of the animal with dental cement. All experiments were started seven days after implantation. ZD7288 (Sigma) or SQ22536 (MCE) was administered after testing baseline pain. ZD7288 (1 ng/nl, 200 nl per side) or SQ22536 (205.22 ng/nl, 200 nl per side) was infused at a rate of 200 nl per min into the VPL. The mice in the control group were intracranially injected with the same volume of ACSF. Mice were euthanized after all behavioral tests, and fluorescent image acquisition with DAPI (4′,6-diamidino-2-phenylindole) staining was performed with the Zeiss LSM 880 confocal microscope. Data from mice with incorrect injection sites were excluded from analysis.

***In vivo* optogenetic manipulations.** For mice expressing eNpHR3.0 or ChR2 in the VPL, optical fiber cannula (the core of 200 μm, Newdoon) were implanted 0.2 mm above the targeting site. Optical fiber cannula was secured to the skulls of mice with screws and dental cement. Chronically implantable fibers (diameter of 200 μm, Newdoon) were connected to a laser generator using optic fiber sleeves. The delivery of a 5-min pulse of blue light (473 nm, 2–5 mW, 20 Hz) or yellow light (594 nm, 5–8 mW, constant) was controlled using a Master-8 pulse stimulator (A.M.P.I.).

***In vivo* multi-channel electrode recordings.** Mice were anesthetized and secured on the stereotaxic apparatus. A custom-made microdrive array attaching 4-8 tetrodes was implanted into the VPL or S1HL. A tetrode was composed of four twisted fine nichrome wires (13 µm, California Fine Wire). The tetrode/optrode was fixed on the brain skull with four screws and dental cement and the mice with a tetrode array were single-housed. Mice were allowed to adapted to having the cables and headstages plugged into the 32-channel connector (Omnetics Connector) for several days prior to recordings. Signals in VPL or S1HL were recorded after at least 4 days of recovery from the surgery, and mice were habituated to cables connected to the electrode on their heads before recording. The subject mice were placed in a cylindrical box wrapped in copper mesh and allowed to move freely without any disturbance, and multichannel electrical signals were recorded throughout the period. Spikes were digitized at 40 kHz, bandpass filtered at 300 to 5,000 Hz, and stored in a computer with NeuroStudio software for further analysis. Data were analyzed using NeuroExplorer 4 (Plexon) and were exported to Offline Sorter 4 (Plexon) for spike sorting. Units with a signal-to-noise ratio smaller than 2 were excluded from analysis. Principal component analysis and threshold crossing were applied to automatically identify waveforms into individual units. Units with inter-spike intervals longer than the refractory periods (1 ms) were determined to be isolated and included in further analysis. Well-isolated units (L ratio < 0.2, isolation distance > 15) were classified into narrow-spiking interneurons or wide-spiking putative pyramidal neurons using an unsupervised clustering algorithm in terms of a κ-means method (3). The algorithm separated each neuron in terms of three-dimensional parameters, including the mean firing rate, the half-valley width, and half-spike width (trough to peak duration) at baseline. Spikes with a slower firing rate, longer half-valley width and longer half-spike width were distinguished to be putative pyramidal neurons. Most of the pyramidal neurons in the somatosensory cortex are known to be glutamatergic neurons.

For in vivo optogenetic tagging of VPL glutamatergic neurons, *CaMKII-Cre* mice were unilaterally injected with AAV-DIO-ChR2-mCherry in the VPL. Three weeks later, optrodes were implanted at the same coordinates where the virus was injected. The optrode was constructed by surrounding an optical fiber (200 μm, Newdoon) with four tetrodes, whereby the tip of tetrodes was 200 mm longer than the fiber. For optical identification of VPL^Glu^ neurons, blue-light pulses (470 nm, 2-ms duration, 20 Hz) were delivered at the end of each recording session at high frequencies. After light-pulse illumination, the units were considered light-responsive when they exhibited time-locked spiking with low jitter (<2 ms), short first-spike latency (<3 ms) and high reliability (>90%). Only when the waveforms of laser-evoked and spontaneous spikes were highly similar (correlation coefficient >0.9) were they considered to originate from a single neuron.

For in vivo electrophysiology with chemogenetic verification of virus function, C57 mice were unilaterally injected with AAV-CaMKII-mCherry or AAV-CaMKII-hM4Di-mCherry into the VPL and S1HL. Multichannel electrical signals were recorded after injection with CNO. The location of the tetrode was examined in all mice at the conclusion of the experiments, and data obtained from mice in which the tetrode was located outside the desired brain region were discarded.

***In vivo* Ca^2+^ signal recordings.** After adjusting the level of the skull surface in the stereotaxic apparatus, rAAV-Ef1a-CaMKII-GCaMP6m-WPRE-pA (AAV-CaMKII-GCaMP6m, AAV2/9, 2.53 × 10^12^ vg/ml, 200 nl) was injected into the VPL or S1HL of C57 mice at a rate of 50 nl/min and an optic fiber (diameter of 200 μm, Newdoon) was slowly implanted into the VPL and S1HL 0.2 mm above the target site after AAV virus injection. After 3 weeks, a multi-channel fiber photometry device (Inper-C1-3C, Inper) delivered exciting LEDs (410 nm and 470 nm) to excite the GCaMP6m fluorophore and collect the emission through the patch cable (0.37 NA, 200μm, Inper) and the implanted optical fiber. The region of interest around the fiber was drawn out to maintain the average intensity, and the behavioral videos were synchronized with the neuronal calcium signals using TTL pulses during recording. A time stamp was assigned to a single recorded time point to be consistent with a specific time and event during recording. Calcium signals were digitized using a digital signal acquisition board and demultiplexed using a software lock-in amplifier; then, signals were low-pass filtered to 30 Hz and saved to a disk at a rate of 381 samples/sec. Photometry data were next analyzed with the Inper Data Process. The values of fluorescence change (ΔF/F), calculated as (Fsignal− Fbaseline)/Fbaseline × 100, are presented as heatmaps or average plots with the SEM. Fbaseline is the mean of fluorescence signal for 5 seconds prior to the von Frey stimulus, and Fsignal is the fluorescence signal for the entire session.

***In vitro* electrophysiological recordings.**

*Brain slice preparation*. Mice were deeply anesthetized with pentobarbital sodium and then perfused through the left ventricle with ice-cold oxygenated N-methyl-D-glucamine (NMDG)-based artificial cerebrospinal fluid (NMDG ACSF), which contained (in mM) 20 HEPES buffer, 93 NMDG, 25 glucose, 2.5 KCl, 1.2 NaH_2_PO_4_, 30 NaHCO_3_, 0.5 CaCl_2_, 5 Na-ascorbate, 10 MgSO_4_, 3Na-pyruvate, 3 glutathione, and 2 thiourea (pH 7.3–7.4, osmolarity of 300–305 mOsm). The brain was then extracted and sectioned into coronal slices (300μm) using a vibrating microtome system (VT1200s, Leica). All brain slices (including VPL and S1HL) were initially incubated in oxygenated NMDG ACSF at 33°C for 10 min and then recovered in oxygenated N-2-hydroxyethylpiperazine-N'-2-ethanesulfonic acid (HEPES)-buffered ACSF at 28°C for at least 1 h, which contained (in mM) 2.5 KCl, 92 NaCl, 2 CaCl_2_, 1.2 NaH_2_PO_4_, 2 CaCl_2_, 30 NaHCO_3_, 3 Na-pyruvate, 5 Na-ascorbate, 20 HEPES, 2 MgSO_4_, 25 glucose, 2 thiourea, and 3 GSH (pH:7.3‒7.4, osmolarity: 300‒310 mOsm). Brain slices were subsequently transferred into a slice chamber (Warner Instruments) for electrophysiological recording and continuously perfused with oxygenated standard ACSF at 32°C, which contained (in mM) 3 KCl, 20 NaHCO_3_, 129 NaCl, 2.4 CaCl_2_, 1.3 MgSO_4_, 3 HEPES, 1.2 KH_2_PO_4_, and 10 glucose (pH: 7.3‒7.4, osmolarity: 300‒310 mOsm). No Ca^2+^ ACSF was used for recording the HCN2 currents (*I*_h_), which contained (in mM): 120 NaCl, 3 KCl, 2 MgCl_2_, 20 NaHCO_3_, 1.2 KH_2_PO_4_, 3 HEPES, and 10 glucose (pH: 7.3‒7.4, osmolarity: 300‒310 mOsm).

*Whole-cell patch-clamp recordings*. Neurons in the VPL or S1HL were visualized with a water immersion objective (×40) in an infrared-differential interference contrast microscope (BX51Wl, Olympus). A MultiClamp 700B amplifier and pCLAMP10.7 software were applied to acquire electrophysiological signals. After a stable Gigaseal was formed, the capacitance and series resistance were automatically compensated. Whole-cell patch-clamp recordings were performed between 2 and 5 min after break-in. Current-evoked tonic firing and burst firing were recorded in current-clamp mode (*I*_hold_ = 0 pA). The pipettes (5‒8 MΩ) used in the recordings were filling with potassium-gluconate-based internal solution, containing (in mM) 130 K-gluconate, 5 KCl, 2 MgCl_2_, 10 HEPES, 0.6 EGTA, 0.3 Na-GTP and 2 Mg-ATP (pH 7.2, osmolality of 285–290 mOsm). The threshold current of the action potential was defined as the minimum current to elicit an action potential. The recordings were made at least 5 min after establishing a whole cell configuration with a stable resting membrane potential. 0.5% neurobiocytin 488 was added to the intracellular solution and immunofluorescent staining was performed for cell type identification in some experiments.

*Spike, sag and I_h_ current recording*. Current-evoked tonic and burst firing were recorded under current-clamp mode (*I*_hold_ = 0 pA) and were obtained with a series of depolarized (from 0 pA to 320 or 400 pA, 20 pA/step, 500 ms) or hyperpolarized (from −10 pA to −300 pA, −10 pA/step, 500 ms) currents delivered to neurons. The sag amplitude was measured as the difference between the peak membrane potential of the hyperpolarization (V_peak_) and the steady-state potential (V_ss_) near the end of the current steps. Data were only collected from neurons with a resting membrane potential lower than −50 mV. We plotted the current–voltage (*I-V*) curve to describe changes in the membrane potential as a function of intracellularly injected currents (−10 to −60 pA, −10 pA/step), and the curve’s slope was derived from the linear portion of the curve. The slope of the *I-V* curve was defined as the input resistance of the cell membrane, and the rheobase for current-evoked firing (tonic or burst) was defined as the minimal strength of current injection required to elicit at least one or two spikes. To directly separate *I*_h_ currents of VPL^Glu^ neurons, the membrane voltage was clamped at -50 mV with 6 s-long voltage steps through −50 mV to −130 mV. During recordings, the TEA-Cl (10 mM), potassium channel blocker 4-aminopyridine (4-AP, 4 mM), BaCl_2_ (200 μM) and tetrodotoxin (TTX, 1 μM) were added into the no Ca^2+^ ACSF for isolated the *I*_h_ currents.

*Light-evoked response*. Light-evoked action potentials or light-induced suppression of firing were recorded in ChR2-expressing or eNpHR3.0-expressing VPL^Glu^ neurons while pulsed blue light (473 nm, 20 ms) or constant yellow ligth (589 nm, 400 ms) were delivered through an optical fiber positioned 0.2 mm above the VPL brain slices. To verify the synaptic functionality of VPL^Glu^→S1HL^Glu^ neurons, light-evoked excitatory postsynaptic currents (EPSCs) were recorded in S1HL^Glu^ neurons with a holding potential of −70 mV while photostimulating (473 nm, 20 ms, 2 Hz) ChR2^+^ VPL^Glu^ terminals in the S1HL. Paired pulse ratio (PPR) of light-evoked (473 nm, pulse width 10 ms, 50 ms interstimulus interval) EPSCs was defined as the ratio of the amplitude of the second EPSC to the first one. The TTX (1 μM), 4-AP (4 mM), and AMPA receptor antagonist 6,7-dinitroquinoxaline-2,3(1H,4H)-dione (DNQX, 20 μM) were used to verify monosynaptic excitatory glutamatergic projections. Drugs used for electrophysiology were dissolved in ACSF to aliquot at 1000× final concentration and stored at −20°C before use. Clampfit software v.10.7 (Axon Instruments) was used to conduct offline analyses of data from in vitro electrophysiological recordings. The relevant information of drugs is reported in Supplemental Table 2.

***In vivo* two-photon calcium imaging.**

*Cranial window surgery*. Mice were deeply anesthetized with an intraperitoneal injection of pentobarbital and then provided with the antiphlogistic carprofen (6 mg per kg, subcutaneously), dexamethasone (25 mg per kg, subcutaneously) and enrofloxacin (125 mg per kg, intraperitoneally) before surgery. To visualize the activity of S1HL^Glu^ neurons, C57 mice were injected with AAV-CaMKII-GCaMP6f-WPRE-pA (AAV-CaMKII-GCaMP6f, AAV2/9, 5.45 × 10^12^ vg/ml) into the S1HL; 1.2% agarose was dropped to cover the dura surface, and a round coverslip (3 mm, Bellco Glass Inc.) was then inserted to fit into the craniotomy and cemented to the skull using glue (Vetbond tissue adhesive, 3M). Custom-designed stainless steel headbars were attached to the skull screw, and dental cement and tissue glue were used to affix both the coverglass and headbar to the skull. The mice were then administered carprofen (6 mg/kg, subcutaneously) lasting 7 days after surgery.

*Two-photon imaging*. Three weeks after the operation, mice were adapted to the headplate holder and the imaging environment for 15 mins each day 3 days before imaging. An upright two-photon microscope (FVMPE-RS, Olympus) in frame-scan mode was applied for awake calcium imaging. Real-time images were acquired using FV30S-SW (Olympus) and a macro water objective lens (×25/0.8 NA) equipped with an infrared laser with an excitation wavelength of 920 nm. For each slice, 300 frames were obtained at a frequency of 1.5 Hz for 256×256 pixels in the x–y plane. The typical average power (20 – 30 mW) was applied to image GCaMP6f-expressing neurons in the S1HL.

*Data processing and analysis* Time-series data were exported to ImageJ for further analysis. Imaging data were corrected for mechanical drift using TurboReg, a plugin of ImageJ available for download online (http://bigwww.epfl.ch/thevenaz/turboreg/). Individual neurons were distinguished from the calcium images using custom MATLAB scripts that implemented component extraction in terms of describing the spatial footprint (shape and location) and the activity trace of the objective. Manual inspection of individual neurons provided quality control. Fluorescence signal time series with ΔF/F traces were automatically analyzed as the fluorescence change based on the baseline fluorescence of components. Calcium signal traces (ΔF/F > 0.5) in the soma were identified as significant calcium events using the MATLAB-based open-source tool CaImAn (<https://github.com/flatironinstitute/CaImAn-MATLAB>), which has previously been used for similar analyses (4). The frequency of GCaMP6f signals in neuronal soma was calculated using the CaImAn‘‘findpeaks’’ function.

**Immunohistochemistry and imaging.** Mice were deeply anesthetized with an intraperitoneal injection of pentobarbital sodium and then perfused with ice-cold 0.9% saline followed by 4% PFA through the left ventricle. The brains were extracted and soaked in 4% PFA at 4°C overnight and then immersed in 20% and 30% sucrose solution for dehydration until they sank. Brains were cut into coronal slices with a thickness of 40 μm using a cryostat microtome system (Leica CM1860) at −20°C. Brain slices were soaked in antifreeze and stored at −20°C. For immunofluorescence, the sections were first washed 3 times with phosphate-buffered saline (PBS) for 10 min and then were incubated with blocking buffer (0.3% Triton X-100, 10% donkey serum in PBS) at room temperature for 1 h. The sections were next treated with primary antibodies in PBS with donkey serum and 3% Triton X-100 at 4°C overnight. Finally, slices were washed 3 times with PBS for 10 min and incubated with secondary antibodies in PBS with 3% Triton X-100 for 1.5 hr at room temperature. After rinsing, the slices were incubated in 4,6-diamidino-2-phenylindole (DAPI; 1:2,000; Sigma) at the last stage. The slices were scanned and imaged using a Zeiss LSM880 microscope to visualize the fluorescence signals.

For post hoc immunofluorescence, slices were collected after whole-cell recording, in which the patched neurons were labeled with neuronbictin-488, were extracted and soaked in 4% PFA at 4°C overnight and then immersed in 20% and 30% sucrose solution for dehydration until they sank. Then, slices were washed 3 times in PBS for 10 minutes, followed by and then were incubated with blocking buffer (0.3% Triton X-100, 10% donkey serum in PBS) at room temperature for 1 h. The slices were next treated with primary antibodies- anti-Glutamate (1:100, rabbit, Sigma) in PBS with donkey serum and 3% Triton X-100 at 4°C for three days. Finally, slices were washed 3 times with PBS for 10 min and incubated with secondary antibodies in PBS with 3% Triton X-100 for 1.5 h at room temperature. The slices were scanned and imaged using a Zeiss LSM880 microscope to visualize the fluorescence signals. The relevant information of antibodies is reported in Supplemental Table 2.

**Western blotting.** The VPL tissues were quickly obtained from 300 μm-thick slices taken on the vibratome. To extract total protein, the tissues were homogenized in ice-cold RIPA buffer, which contained 50 mM Tris-HCl (pH 7.6), 1% Triton X-100, 150 mM NaCl, 0.1% SDS, a protease inhibitor cocktail, and 0.5% sodium deoxycholate. Then, the proteins were obtained by centrifuging at 12,000 g at 4°C for 15 min, and the protein concentrations were measured by BCA assay. In each lane, 10–20 μg protein was separated via 6% SDS-PAGE gel electrophoresis and then transferred to polyvinylidene difluoride (PVDF) membranes (Bio-Rad). After blocking with 5% skim milk, the membranes were incubated with primary antibodies, including HCN2 (1:1000, Thermo), HCN3 (1:500, Thermo), HCN4 (1:500, Thermo) and beta-actin (1:1000, Absin), at 4°C overnight and then secondary antibody peroxidase-labeled goat anti-mouse (1:5000, Jackson) at room temperature for 90 min. All the protein bands were visualized using high-sensitivity ECL reagent (GE Healthcare) and analyzed using ImageJ software. The relevant information of antibodies is reported in Supplemental Table 2.

**cAMP measurement.** The VPL tissues were quickly extracted from 300 μm-thick slices collected by vibratome. cAMP levels were measured using a cAMP ELISA Kit (D770001-0096, Sangon Biotech, shanghai, China) following the manufacturer’s instructions. Briefly, the microtiter plate provided in the kit was pre-coated with goat anti-mouse polyclonal antibody. cAMP was extracted with 0.1M HCl, and the extracts were added to the appropriate microtiter plate wells with an antibody specific to cAMP and horseradish peroxidase conjugated cAMP. After incubation for about 10 min, the reaction was read at 450 nm. The relevant information of antibodies is reported in Supplemental Table 2.

**Statistical analysis.** All data were calculated based on the results of our experiments. Mice were randomly assigned to each treatment, and the analyses were performed by an assistant blinded to the treatment assignment. GraphPad Prism 8 (GraphPad Software, Inc., USA) was used for statistical analysis and graphing. Clampfit software v.10.7 (Axon Instruments) was used to conduct offline analyses of data from in vitro electrophysiological recordings. The relevant information of software is reported in Supplemental Table 2. Paired or unpaired two-tailed Student’s t-tests were applied for statistical comparisons between two groups. One-way or two-way analysis of variance (ANOVA) followed by Bonferroni’s multiple comparisons test was used for analysis with multiple groups. Data are presented as the mean ± SEM, and *P* < 0.05 was considered to be significant. The sample sizes, statistical tests, and other relevant information of statistical analysis are reported in Table S1.

**References**

1. Madisen, L, Zwingman, TA, Sunkin, SM*, et al.* A robust and high-throughput Cre reporting and characterization system for the whole mouse brain. *Nat Neurosci*. 2010; **13**(1): 133-40.

2. Dragatsis, I, Zeitlin, S. CaMKIIalpha-Cre transgene expression and recombination patterns in the mouse brain. *Genesis*. 2000; **26**(2): 133-5.

3. Xu, H, Liu, L, Tian, Y*, et al.* A Disinhibitory Microcircuit Mediates Conditioned Social Fear in the Prefrontal Cortex. *Neuron*. 2019; **102**(3): 668-82 e5.

4. Giovannucci, A, Friedrich, J, Gunn, P*, et al.* CaImAn an open source tool for scalable calcium imaging data analysis. *Elife*. 2019; **8**.

**Supplementary Figure titles and legends**

**Fig. S1 Increased c-Fos expression of VPL^Glu^ neurons in a mouse model of chronic inflammatory pain induced by CFA injection.**

(a) Schematic of the animal model of CFA. BL, baseline.

(b) Time course of changes in response threshold to mechanical force via von Frey test in CFA mouse model of chronic pain.

(c) Images showing the c-Fos expression in thalamic neurons in saline and CFA 3D mice. Scale bar, 200 µm. The insert depicts the area shown in the white box of the VPL. Scale bar, 20 µm.

(d) Images (left) and statistics data (right) showing that c-Fos-postive neurons within VPL were mainly co-labeled with glutamate immunofluorescence. Scale bar, 10 µm. For detailed statistical information, see Table S1.

**Fig. S2 Validation of glutamate antibody specificity and sensitivity.**

(a) Schematic for viral injection.

(b) Representative images of the injection site (left) and mCherry^+^ neurons co-localized with EGFP^+^ neurons within the VPL (right). Scale bars, 200 µm (left) or 10 µm (right).

(c) Statistical analysis of co-localization between mCherry^+^ neurons and EGFP^+^ neurons.

(d) Representative images showing mCherry^+^EGFP^+^ co-labeled neurons co-localized with glutamate antibody (purple) within the VPL (right). Scale bars, 10 µm.

(e) Statistical analysis of co-localization between mCherry^+^ and EGFP^+^ co-labeled neurons and glutamate antibody.

**Fig. S3 Optogenetic tagging of glutamatergic neurons in the VPL.**

(a) Schematic of virus injection and the recording configuration.

(b) Images showing the injection site (left) and mCherry-positive neurons (red) co-localized with glutamate (Glu, green) immunofluorescence (right) within the VPL. Scale bars, 200 µm (left) or 10 µm (right).

(c) Statistical data showing mCherry^+^ neurons were mainly co-localized with glutamate immunofluorescence.

(d) Left: example recording of spontaneous and light-evoked spikes from VPL^Glu^ neurons; Right: overlay of light-evoked (blue) and averaged spontaneous (red) spike waveforms from the example unit.

(e) Recorded light-sensitive neurons were classified as wide-spiking putative glutamatergic cells (n = 23) according to firing rate, half width and trough to peak duration of the spike.

**Fig. S4 Increased excitability of VPL^Glu^ neurons in a mouse model of chronic inflammatory pain induced by CFA injection.**

(a-c) Sample traces (a) and summarized data of firing rates (b), and rheobase of the spike (c) in VPL^Glu^ neurons recorded from sham and CFA 3D mice.

(d and e) Summarized data of RMP (d) and R_in_ (e) of VPL^Glu^ neurons in saline and CFA 3D mice.

(f and g) Representative traces (f) and statistical data (g) showing the spontaneous spikes recorded from VPL^Glu^ neurons in saline and CFA 3D mice.

(h and i) Heatmaps (h) and the mean data (i) showing the change of VPL-Glu^GCaMP6m^ signals in saline and CFA 3D mice. The colored bar at the right in (h) indicates ΔF/F (%).

All data are mean ± SEM. **P* < 0.05, ***P* < 0.01, n.s., not significant. For detailed statistics information, see Table S1.

**Fig. S5 Chemogenetic inhibition of VPL^Glu^ neurons reverses allodynia in CFA 3D mice.**

(a) Schematic of the experimental procedure.

(b and c) Representative traces (b) and summarized data (c) of spontaneous spikes in VPL^Glu^ neurons of saline and CFA 3D mice infected with mCherry or hM4Di-mCherry within VPL.

(d) Effects of chemogenetic inhibition of VPL^Glu^ neurons on the pain threshold in CFA 3D mice.

All data are mean ± SEM. **P* < 0.05, ***P* < 0.01, ****P* < 0.001. For detailed statistics information, see Table S1.

**Fig. S6 Immunofluorescence staining of HCN2, HCN3 and HCN4 channels in the VPL.**

(a-c) Reprehensive images showing the HCN2 (a), HCN3 (b) and HCN4 (c) expressions in thalamic neurons. Scale bar, 200 µm. The magnification depicts the area shown in the white box of the VPL. Scale bar, 50 µm.

**Fig. S7 Western blotting of VPL lysates with antibodies against HCN2, HCN3, HCN4 and β-actin in saline and CFA 3D mice.**

(a) Western blots of VPL lysates to detect HCN2 protein levels in Saline and CFA 3D mice.

(b) Western blotting of VPL lysates to detect HCN3 protein levels in saline and CFA 3D mice.

(c) Western blotting of VPL lysates to detect HCN4 protein levels in saline and CFA 3D mice.

All data are mean ± SEM. **P* < 0.05, ***P* < 0.01. n.s., no significant. For detailed statistics information, see Table S1.

**Fig. S8 Electrophysiological recordings of *I*_h_ currents in VPL^Glu^ neurons.**

(a) Images showing HCN2 channels (red) co-localized with glutamate immunofluorescence (Glu, green) within the VPL. Scale bar, 10 µm.

(b) Representative traces of *I*_h_ currents recorded from VPL^Glu^ neurons blocked by administration of ZD7288.

(c) Current density (pA*/*pF) of *I*_h_ is plotted against the voltage step in naive mice treat with ACSF or ZD7288.

All data are mean ± SEM. ****P* < 0.001. For detailed statistics information, see Table S1.

**Fig. S9 Functional upregulation of HCN2 channels in CFA 3D mice.**

(a-d) Sample traces (a) and summary data of the spike in burst (b), the summarized data of burst spike rheobase (c) and sag amplitude (d) in VPL^Glu^ neurons recorded from saline and CFA 3D mice.

(e) Representative *I*_h_ current recorded from VPL^Glu^ neurons in saline and CFA 3D mice.

(f) Current density (pA*/*pF) of *I*_h_ is plotted against the voltage step.

(g) Schematic of the experimental procedure for mice with cannula implanted, CFA treatment and behavioral tests.

(h and i) Sample traces (h) and summarized data (i) of firing rates recorded in VPL^Glu^ neurons of CFA 3D mice treated with ACSF or ZD7288.

(j) Summarized data the burst spike rheobase of VPL^Glu^ neurons in CFA 3D mice treated with ACSF or ZD7288.

(k and l) Sample traces (k) and summarized data (l) of the spike in burst recorded from VPL^Glu^ neurons in CFA 3D mice treated with ACSF or ZD7288.

(m-p) Statistical data of the rheobase of the burst spike (m), sag amplitude (n), RMP (o) and R_in_ (p) of VPL^Glu^ neurons in CFA 3D mice treated with ACSF or ZD7288.

(q) Effects of pharmacological inhibition of HCN2 channels in the VPL on the pain threshold of saline and CFA 3D mice.

All data are mean ± SEM. **P* < 0.05, ***P* < 0.01, ****P* < 0.001, n.s., not significant. For detailed statistics information, see Table S1.

**Fig. S10 VPL^Glu^ neurons projecting onto S1HL^Glu^ neurons.**

(a) Schematic of virus injection.

(b) Representative images of mCherry^+^ fibers in the S1HL of C57 mice with VPL injection of AAV-CaMKII-ChR2-mCherry. Scale bars, 100 µm. mCherry^+^ fibers were not found in ACC, mPFC, IC.

(c) Schematic of the Cre-dependent retrograde trans-monosynaptic rabies virus tracing strategy.

(d) Representative images showing input neurons in the PO, ZI, contralateral S1HL, and S2. Scale bars, 200 µm

(e) The expression of DsRed^+^ neurons at different bregma sites in the thalamus of *CaMKII-Cre* mice. Scale bar, 200 µm.

(f) Images showing that DsRed^+^ neurons (red) co-labled with HCN2 (purple) and glutamate (green) and immunofluorescence. Scale bar, 20 µm.

ACC, anterior cingulate; mPFC, medial prefrontal cortex; IC, insular cortex; PO, posterior thalamic nucleus; ZI, zona incerta; S2, secondary somatosensory cortex.

**Fig. S11** **Optogenetic tagging of glutamatergic neurons in the S1HL.**

(a) Schematic of virus injection and recording configuration.

(b) Images showing the injection site within the S1HL. Scale bars, 200 µm.

(c) Images (left) and statistical data (right) showing mCherry-positive neurons (red) co-localized with glutamate (Glu, green) immunofluorescence (right). Scale bar, 10 µm.

(d) Left: example recording of spontaneous and light-evoked spikes from S1HL^Glu^ neurons; Right: overlay of light-evoked (blue) and averaged spontaneous (red) spike waveforms from the example unit.

(e) Recorded light-sensitive neurons were classified as wide-spiking putative glutamatergic cells (n = 30) according to firing rate, half width and trough to peak duration of the spike.

**Fig. S12 Enhanced activity of S1HL^Glu^ neurons in CFA 3D mice.**

(a) Schematic of virus injection and recording configuration.

(b and c) Representative traces (b) and summarized data (c) of the PPR of light-evoked EPSCs in the S1HL^Glu^ neuron in saline and CFA 3D mice.

(d) Schematic of electrophysiological recording in the S1HL of freely moving mice. The enlargement shows the multichannel tetrode.

(e and f) Representative traces (e) and statistical data (f) showing the spontaneous spikes recorded from S1HL^Glu^ neurons in saline and CFA 3D mice.

(g) Schematic of S1HL^Glu^ injection of AAV-CaMKII- GCaMP6m-GFP in C57 mice.

(h and i) Heatmaps (h) and the mean data (i) showing the change of S1HL-Glu^GCaMP6m^ signals in saline and CFA 3D mice. The colored bar at the right in (h) indicates ΔF/F (%).

(j) Schematic of the *in vivo* two-photon (2P) calcium image in head-restrained C57 mice with AAV-CaMKII-GCaMP6f-GFP expressing in S1HL^Glu^ neurons.

(k and l) Representative traces (k) and numbers matching spontaneous ΔF/F time series traces (l) showing that the fluorescence intensity of GCaMP6^+^ S1HL^Glu^ neurons. Scale bar, 50 µm in (k).

(m and n) Representative traces (m) and numbers matching spontaneous ΔF/F time series traces (n) showing that the fluorescence intensity of GCaMP6^+^ S1HL^Glu^ neurons is increased in CFA 3D mice compared with saline mice. Scale bar, 50 µm in (m).

(o and p) Statistical data showing the fluorescence intensity (o) and calcium influx events (p) of GCaMP6m-expressing S1HL^Glu^ neurons in saline and CFA 3D mice.

All data are mean ± SEM. ****P* < 0.001. For detailed statistics information, see Table S1.

**Fig. S13 Chemogenetic inhibition of VPL^Glu^ neurons reduces S1HL^Glu^ neuronal activity in mouse models of chronic pain.**

(a) Schematic of the experimental procedure.

(b and c) Representative images (b) and statistical analysis (c) showing the distribution of c-Fos-positive neurons in the S1HL of sham and SNI 7D mice injected with AAV-CaMKII-mCherry or AAV-CaMKII-hM4Di-mCherry in the VPL. Scale bar, 200 µm.

(d) Schematic of the experimental procedure.

(e and f) Representative images (e) and statistical analysis (f) showing the distribution of c-Fos-positive neurons in the S1HL of saline and CFA 3D mice injected with mCherry or hM4Di-mCherry in the VPL. Scale bar, 200 µm.

(g) Schematic of the experimental procedure for mice with virus injection, CFA treatment, and multi-channel electrode recording.

(h and i) Representative traces (h) and summarized data (i) of spontaneous spikes in S1HL^Glu^ neurons of sham and SNI 7D mice infected with mCherry or hM4Di-mCherry within VPL.

(j) Effects of optogenetic inhibition of VPL^Glu^ neuronal fibers in the S1HLon pain thresholds of SNI 7D mice.

All data are mean ± SEM. **P* < 0.05, ***P* < 0.01, ****P* < 0.001. For detailed statistics information, see Table S1.

**Fig. S14 Optical activation of VPL^Glu^ neuronal terminals in the S1HL induces allodynia in mice.**

(a) Schematic of optogenetic experiments in C57 mice.

(b) Left: images showing that the mCherry^+^ fibers within S1HL with VPL injection of AAV-CaMKII-ChR2-mCherry or AAV-CaMKII-mCherry. The background color (green) is the immunofluorescence staining of glutamate (Glu). Right: typical images showing the region in the white box in the S1HL. Scale bars, 200 µm (Left) or 10 µm (Right).

(c) Effects of optogenetic activation of VPL^Glu^ neuronal fibers in the S1HL on the pain thresholds of normal mice.

All data are mean ± SEM. **P* < 0.05. For detailed statistics information, see Table S1.

**Fig. S15 The infection of AAV-DIO-mCherry-shRNA(HCN2) virus in the VPL of *CaMKII-Cre* mice.**

(a) Schematic of viral injection.

(b and c) Representative imagings showing AAV-DIO-mCherry-shRNA(HCN2) infected neurons at different bregma sites in the VPL. Scale bar, 200 µm.

**Fig. S16 Functional knockdown of HCN2 channels in the VPL^Glu^→S1HL^Glu^ circuit rescues allodynia in CFA 3D mice.**

(a) Schematic of the experimental procedure.

(b and c) Images (b) and statistical analysis (c) showing the distribution of c-Fos-positive neurons in the S1HL of saline and CFA 3D mice infused with ACSF or ZD7288 in the VPL. Scale bar, 200 µm.

(d) Schematic of the experimental procedure for mice with virus injection in the VPL, CFA treatment and behavioral tests.

(e) Effects of downregulating HCN2 channels in VPL^Glu^ neurons on the pain threshold of SNI 7D mice.

(f) Schematic of the fiber photometry recordings in CFA 3D mice. Ca^2+^ signals transients were recorded from GCaMP6m-expressing S1HL^Glu^ neurons in *CaMKII-Cre* mice with VPL infusion of AAV-DIO-mCherry-shRNA (HCN2) or AAV-DIO-mCherry-shRNA(scramble).

(g and h) The heatmaps (g) and the mean data (h) showing the change of S1HL-Glu^GCaMP6m^ signals in CFA 3D mice infusion of AAV-DIO-mCherry-shRNA (HCN2) or AAV-DIO-mCherry-shRNA(scramble) with the VPL. The colored bar at the right in (g) indicates ΔF/F (%).

(i) Effects of downregulating HCN2 channels in VPL^Glu^→S1HL^Glu^ pathway on the pain threshold in CFA 3D mice.

All data are mean ± SEM. ****P* < 0.001. For detailed statistics information, see Table S1.

**Fig. S17 HCN2 channel overexpression in the VPL^Glu^→S1HL^Glu^ circuit induces allodynia in normal mice.**

(a) Schematic for virus injection.

(b) Representative images of the AAV-DIO-HCN2-3xflag injection site in the VPL. Scale bar, 200 µm.

(c) Top: schematic illustration of the strategy for HCN2 channel overexpression with AAV-DIO-HCN2-3xflag; Bottom: Images showing neurons (red) co-localized with glutamate immunofluorescence (Glu, green) within the VPL. Scale bar, 10 µm.

(d) Statistical analysis of virus-infected neuron co-localization with glutamate immunofluorescence.

(e) Western blots of lysates of 3xflag-infected (AAV-control) and HCN2-infected (AAV-HCN2) VPL tissue in *CaMKII-Cre* mice using antibodies against HCN2 or β-actin.

(f) Representative traces of *I*_h_ currents recorded from VPL^Glu^ neurons recorded in *CaMKII-Cre* mice with VPL infusion of AAV-DIO-HCN2-3xflag or AAV-DIO-3xflag.

(g) Current density *(*pA*/*pF*)* of *I*_h_ is plotted against the voltage step.

(h-j) Sample traces (h) and summarized data of firing rates (i), and rheobase of the spike (j) recorded from VPL^Glu^ neurons.

(k-m) Sample traces (k) and summarized data of the burst spike (l), and the rheobase of the burst spike (m) recorded in VPL^Glu^ neurons.

(n-p) Summarized data of the sag amplitude (n), RMP (o) and R_in_ (p) recorded from VPL^Glu^ neurons.

(q) Representative images showing 3xflag-infected neurons (red) co-localized with glutamate immunofluorescence. Scale bar, 20 µm.

(r) Schematic of fiber photometry recordings in non-model mice. Ca^2+^ signal transients were recorded in GCaMP6m-expressing S1HL^Glu^ neurons of *CaMKII-Cre* mice with VPL infusion of AAV-DIO-HCN2-3xflag or AAV-DIO-3xflag.

(s and t) Heatmaps (s) and mean changes (t) in S1HL-Glu^GCaMP6m^ signals in *CaMKII-Cre* mice. The colored bar at the right in (s) indicates ΔF/F (%).

(u) Schematic of the experimental procedure.

(v) Effects of VPL^Glu^ neuron-specific HCN2 channel upregulation on the mechanical pain thresholds of C57 mice.

(w) Schematic for optogenetic experiments in C57 mice.

(x) Representative images of the injection site in the VPL. Scale bar, 200 µm.

(y) Effects of upregulating HCN2 channels in the VPL^Glu^→S1HL^Glu^ pathway on mechanical pain threshold in C57 mice.

All data are means ± SEM. ***P* < 0.01, ****P* < 0.001. n.s., not significant. For detailed statistical information, see Table S1.

**Fig. S18 Inhibition of cAMP attenuates VPL^Glu^ neuronal activity and relieves allodynia in SNI 7D mice.**

(a) Enzyme-linked immunosorbent assays of cAMP levels in VPL lysates of sham and SNI 7D mice.

(b) Schematic for cannula implantation, SNI model establishment, and behavioral tests in mice.

(c) Representative traces of *I*_h_ currents recorded in VPL^Glu^ neurons in the presence of cAMP inhibitor SQ22536 or ACSF.

(d) Current density *(*pA*/*pF*)* of *I*_h_ is plotted against voltage steps in SNI 7D mice treated with ACSF or SQ22536.

(e and f) Sample traces (e) and summarized data (f) of firing rates recorded in VPL^Glu^ neurons of SNI 7D mice treated with ACSF or SQ22536.

(g) Summarized data of tonic spike rheobase in VPL^Glu^ neurons of SNI 7D mice treated with ACSF or SQ22536.

(h and i) Sample traces (h) and summarized data (i) of the spikes in bursts recorded in VPL^Glu^ neurons.

(j-m) Summarized data of burst spike rheobase (j), sag amplitude (k), RMP (l) and R_in_ (m) in VPL^Glu^ neurons of SNI 7D mice treated with ACSF or SQ22536.

(n) Mechanical pain threshold in SNI 7D mice with pharmacological inhibition of cAMP in the VPL.

All data are means ± SEM. **P* < 0.05, ***P* < 0.01, ****P* < 0.001, n.s., not significant. For detailed statistical information, see Table S1.

**Fig. S19 Inhibition of cAMP attenuates VPL^Glu^ neuronal activity and relieves allodynia in CFA 3D mice.**

(a) Enzyme-linked immunosorbent assays of cAMP levels in VPL lysates of sham and CFA 3D mice.

(b) Schematic for experimental procedure.

(c) Representative traces of *I*_h_ currents recorded in VPL^Glu^ neurons in the presence of cAMP inhibitor SQ22536 or ACSF.

(d) Current density (pA*/*pF) of *I*_h_ is plotted against voltage steps in naive mice treated with ACSF or SQ22536.

(e and f) Sample traces (e) and summarized data (f) of firing rates recorded in VPL^Glu^ neurons of CFA 3D mice treated with ACSF or SQ22536.

(g) Summarized data of tonic spike rheobase in VPL^Glu^ neurons of CFA 3D mice.

(h and i) Sample traces (h) and summarized data (i) of the spikes in bursts recorded in VPL^Glu^ neurons of CFA 3D mice treated with ACSF or SQ22536.

(j-m) Summarized data of burst spike rheobase (j), sag amplitude (k), RMP (l) and R_in_ (m) in VPL^Glu^ neurons of CFA 3D mice.

(n) Mechanical pain threshold in CFA 3D mice with pharmacological inhibition of cAMP in the VPL.

All data are means ± SEM. **P* < 0.05, ***P* < 0.01, ****P* < 0.001, n.s., not significant. For detailed statistical information, see Table S1.

**Fig. S20 Functional upregulation of HCN2** **channels enhances thalamocortical VPL^Glu^→S1HL^Glu^ circuit activity to promote allodynia in mice.**

Spared nerve injury (SNI) or complete Freund’s adjuvant (CFA) to the left hindpaws of mice lead to enhanced neuronal activity of contralateral VPL^Glu^ neurons via increased HCN2 channel function. Subsequently, the excitability of S1HL^Glu^ neurons will be elevated by receiving the increased inputs from the elevated VPL^Glu^ neurons. These alterations presumably cause chronic pain of injury hindpaws of mice, which could be alleviated by knockdown of HCN2 channel in VPL^Glu^ neurons.

**Video S1.**

Optical-fiber-based Ca^2+^ signal recordings of S1HL^Glu^ neurons evoked by 0.07-g von Frey filament stimuli on the injury hindpaws of sham and SNI 7D mice.

**Video S2.**

Optical-fiber-based Ca^2+^ signal recordings of S1HL^Glu^ neurons evoked by 0.07-g von Frey filament stimuli on the injury hindpaws of SNI 7D mice with HCN2 channel knockdown in the VPL.

**Video S3.**

Optical-fiber-based Ca^2+^ signal recordings of S1HL^Glu^ neurons evoked by 0.07-g von Frey filament stimuli on the contralateral hindpaws of normal mice with HCN2 channel overexpression in the VPL.

**Table S1. Statistical analyses related to Figs. 1-6 and Figs. S1-19.**

| Figure | Conditions (sample size) | | Analysis | *P* value | *t* or *F* value |
| --- | --- | --- | --- | --- | --- |
| Fig. 1b | Sham (n = 9) | SNI (n = 9) | Two-way RM ANOVA  with Bonferroni post hoc analysis | *P* < 0.0001 | *F*(1, 16) = 944.0 |
|  | 3D | |  | *P* < 0.0001 |  |
|  | 7D | |  | *P* < 0.0001 |  |
|  | 14D | |  | *P* < 0.0001 |  |
|  | 21D | |  | *P* < 0.0001 |  |
|  | 28D | |  | *P* < 0.0001 |  |
|  | 35D | |  | *P* < 0.0001 |  |
|  | 42D | |  | *P* < 0.0001 |  |
| Fig. 1g | Sham (n = 26) | SNI 7D (n = 24) | Two-way RM ANOVA  with Bonferroni post hoc analysis | *P* = 0.0033 | *F*(1, 48) = 9.593 |
| Fig. 1h | Sham (n = 26) | SNI 7D (n = 24) | Unpaired Student’s t-test | *P* = 0.0226 | *t*(48) = 2.356 |
| Fig. 1i | Sham (n = 26) | SNI 7D (n = 24) | Unpaired Student’s t-test | *P* = 0.0039 | *t*(48) = 3.030 |
| Fig. 1j | Sham (n = 26) | SNI 7D (n = 24) | Unpaired Student’s t-test | *P* = 0.5753 | *t*(48) = 0.5642 |
| Fig. 1m | Sham (n = 21) | SNI 7D (n = 24) | Unpaired Student’s t-test | *P* = 0.0401 | *t*(43) = 2.117 |
| Fig. 2f |  |  | One-way RM ANOVA  with Bonferroni post hoc analysis | *P* = 0.0005 | *F*(2, 69) = 8.390 |
|  | Sham-mCherry (n = 24) | SNI-mCherry (n = 25) |  | *P* = 0.0067 |  |
|  | SNI 7D-mCherry (n = 25) | SNI 7D-hM4Di (n = 23) |  | *P* = 0.0005 |  |
| Fig. 2g | SNI 7D-mCherry (n = 7) | SNI 7D-hM4Di (n = 7) | Two-way RM ANOVA  with Bonferroni post hoc analysis | *P* < 0.0001 | *F*(1, 12) = 78.01 |
|  | SNI 7D | |  | *P* > 0.9999 |  |
|  | Post CNO 0.5 h | |  | *P* = 0.5099 |  |
|  | Post CNO 1.0 h | |  | *P* = 0.1328 |  |
|  | Post CNO 1.5 h | |  | *P* = 0.1875 |  |
|  | Post CNO 2.0 h | |  | *P* = 0.0032 |  |
|  | Post CNO 2.5 h | |  | *P* = 0.0002 |  |
|  | Post CNO 3.0 h | |  | *P* < 0.0001 |  |
|  | Post CNO 3.5 h | |  | *P* > 0.9999 |  |
|  | Post CNO 4.0 h | |  | *P* > 0.9999 |  |
| Fig. 2l | SNI 7D-mCherry (n = 7) | SNI 7D-ChR2 (n = 7) | Two-way RM ANOVA  with Bonferroni post hoc analysis | *P* = 0.1515 | *F*(1, 12) = 2.346 |
|  | Light on | |  | *P* = 0.0174 |  |
| Fig. 3b | Sham (n = 25) | SNI 7D (n = 23) | Two-way RM ANOVA  with Bonferroni post hoc analysis | *P* = 0.0216 | *F*(1, 46) = 5.659 |
| Fig. 3c | Sham (n = 25) | SNI 7D (n = 23) | Unpaired Student’s t-test | *P* < 0.0001 | *t*(46) = 4.449 |
| Fig. 3d | Sham (n = 26) | SNI 7D (n = 25) | Two-way RM ANOVA  with Bonferroni post hoc analysis | *P* < 0.0001 | *F*(1, 49) = 26.63 |
| Fig. 3e | Sham (n = 6) | SNI 7D (n = 6) | Unpaired Student’s t-test | *P* = 0.0082 | *t*(10) = 3.287 |
| Fig. 3f | Sham (n = 5) | SNI 7D (n = 5) | Unpaired Student’s t-test | *P* = 0.6842 | *t*(8) = 0.4219 |
| Fig. 3g | Sham (n = 5) | SNI 7D (n = 5) | Unpaired Student’s t-test | *P* = 0.9570 | *t*(8) = 0.0556 |
| Fig. 3i | Sham (n = 19) | SNI 7D (n = 19) | Two-way RM ANOVA  with Bonferroni post hoc analysis | *P* = 0.0060 | *F*(1,36) = 8.513 |
| Fig. 3m | SNI 7D-ACSF (n = 11) | SNI 7D-ZD7288 (n = 11) | Two-way RM ANOVA  with Bonferroni post hocanalysis | *P* = 0.0279 | *F*(1,20) = 5.623 |
| Fig. 3n | SNI 7D-ACSF (n = 11) | SNI 7D-ZD7288 (n = 11) | Unpaired Student’s t-test | *P* = 0.0303 | *t*(20) = 2.332 |
| Fig. 3p | SNI 7D-ACSF (n = 11) | SNI 7D-ZD7288 (n = 11) | Two-way RM ANOVA  with Bonferroni post hoc  analysis | *P* < 0.0001 | *F*(1,20) = 26.47 |
| Fig. 3q | SNI 7D-ACSF (n = 11) | SNI 7D-ZD7288 (n = 11) | Unpaired Student’s t-test | *P* = 0.0004 | *t*(20) = 4.224 |
| Fig. 3r | SNI 7D-ACSF (n = 11) | SNI 7D-ZD7288 (n = 11) | Two-way RM ANOVA  with Bonferroni post hoc  analysis | *P* < 0.0001 | *F*(1,20) = 45.83 |
| Fig. 3s | SNI 7D-ACSF (n = 11) | SNI 7D-ZD7288 (n = 11) | Unpaired Student’s t-test | *P* < 0.0001 | *t*(20) = 5.703 |
| Fig. 3t | SNI 7D-ACSF (n = 11) | SNI 7D-ZD7288 (n = 11) | Unpaired Student’s t-test | *P* = 0.0046 | *t*(20) = 3.042 |
| Fig. 3u | Sham-ACSF (n = 7) | Sham-ZD7288 (n = 7) | Two-way RM ANOVA  with Bonferroni post hoc analysis | *P* = 0.0798 | *F*(1, 12) = 3.662 |
|  | Sham | |  | *P* = 0.8711 |  |
|  | Post ZD7288 0.5 h | |  | *P* = 0.7630 |  |
|  | Post ZD7288 1.0 h | |  | *P* = 0.6661 |  |
|  | Post ZD7288 1.5 h | |  | *P* > 0.9999 |  |
|  | Post ZD7288 2.0 h | |  | *P* > 0.9999 |  |
|  | Post ZD7288 2.5 h | |  | *P* > 0.9999 |  |
|  | Post ZD7288 3.0 h | |  | *P* > 0.9999 |  |
|  | Post ZD7288 3.5 h | |  | *P* > 0.9999 |  |
|  | Post ZD7288 4.0 h | |  | *P* > 0.9999 |  |
|  | SNI 7D-ACSF (n = 7) | SNI 7D-ZD7288 (n = 7) | Two-way RM ANOVA  with Bonferroni post hoc analysis | *P* = 0.0009 | *F*(1, 12) = 19.36 |
|  | SNI 7D | |  | *P* > 0.9999 |  |
|  | Post ZD7288 0.5 h | |  | *P* > 0.9999 |  |
|  | Post ZD7288 1.0 h | |  | *P* > 0.9999 |  |
|  | Post ZD7288 1.5 h | |  | *P* < 0.0001 |  |
|  | Post ZD7288 2.0 h | |  | *P* < 0.0001 |  |
|  | Post ZD7288 2.5 h | |  | *P* > 0.9999 |  |
|  | Post ZD7288 3.0 h | |  | *P* > 0.9999 |  |
|  | Post ZD7288 3.5 h | |  | *P* > 0.9999 |  |
|  | Post ZD7288 4.0 h | |  | *P* > 0.9999 |  |
| Fig. 4p |  |  | Paired Student’s t-test |  |  |
|  | ACSF (n = 5) | TTX (n = 5) |  | *P* = 0.0001 | *t*(4) = 15.20 |
|  | 4-AP+TTX (n = 5) | 4-AP+TTX+DNQX (n = 5) |  | *P* = 0.0003 | *t*(4) = 12.29 |
| Fig. 5c | Sham (n = 11) | SNI 7D (n = 11) | Unpaired Student’s t-test | *P* < 0.0001 | *t*(20) = 5.387 |
| Fig. 5e | Sham (n = 22) | SNI 7D (n = 39) | Unpaired Student’s t-test | *P* = 0.0100 | *t*(59) = 2.661 |
| Fig. 5k | Sham (n = 124) | SNI 7D (n = 132) | Unpaired Student’s t-test | *P* = 0.0006 | *t*(254) = 4.926 |
| Fig. 5l | Sham (n = 124) | SNI 7D (n = 132) | Unpaired Student’s t-test | *P* = 0.0014 | *t*(254) = 3.239 |
| Fig. 5o |  |  | One-way RM ANOVA  with Bonferroni post hoc analysis | *P* = 0.0044 | *F*(2, 91) = 5.751 |
|  | Sham-mCherry (n = 22) | SNI-mCherry (n = 39) |  | *P* = 0.0084 |  |
|  | SNI-mCherry (n = 39) | SNI-hM4Di (n = 33) |  | *P* = 0.0147 |  |
| Fig. 5q |  |  | Two-way RM ANOVA  with Bonferroni post hoc analysis |  |  |
|  | Sham-mCherry (n = 15) | SNI-mCherry (n = 15) |  | *P* < 0.0001 | *F*(1, 28) = 39.02 |
|  | SNI-mCherry (n = 15) | SNI-hM4Di (n = 15) |  | *P* < 0.0001 | *F*(1, 28) = 74.66 |
| Fig. 5r |  |  | One-way RM ANOVA  with Bonferroni post hoc analysis | *P* < 0.0001 | *F*(2, 42) = 15.93 |
|  | Sham-mCherry (n = 15) | SNI-mCherry (n = 15) |  | *P* = 0.0002 |  |
|  | SNI-mCherry (n = 15) | SNI-hM4Di (n = 15) |  | *P* < 0.0001 |  |
| Fig. 5v | SNI 7D-EYFP (n = 5) | SNI 7D-eNpHR 3.0 (n = 7) | Two-way RM ANOVA  with Bonferroni post hoc analysis | *P* < 0.0001 | *F*(1, 10) = 64.54 |
|  | Light on | |  | *P* = 0.0002 |  |
| Fig. 6c |  |  | One-way RM ANOVA  with Bonferroni post hoc analysis | *P* < 0.0001 | *F*(2, 15) = 118.4 |
|  | Sham-ACSF (n = 6) | SNI 7D-ACSF (n = 6) |  | *P* < 0.0001 |  |
|  | SNI 7D-ACSF (n = 6) | SNI 7D-ZD7288 (n = 6) |  | *P* < 0.0001 |  |
| Fig. 6g |  |  | Two-way RM ANOVA  with Bonferroni post hoc  analysis |  |  |
|  | Sham-ACSF (n = 13) | SNI 7D-ACSF (n = 13) |  | *P* < 0.0001 | *F*(1, 24) = 39.91 |
|  | SNI 7D-ACSF (n = 13) | SNI 7D-ZD7288 (n = 13) |  | *P* < 0.0001 | *F*(1, 24) = 26.00 |
| Fig. 6h |  |  | One-way RM ANOVA  with Bonferroni post hoc analysis | *P* < 0.0001 | *F*(2, 36) = 13.74 |
|  | Sham-ACSF (n = 13) | SNI 7D-ACSF (n = 13) |  | *P* < 0.0001 |  |
|  | SNI 7D-ACSF (n = 13) | SNI 7D-ZD7288 (n = 13) |  | *P* = 0.0002 |  |
| Fig. 6m | AAV-mCherry (n = 5) | AAV-shRNA (n = 5) | Unpaired Student’s t-test | *P* = 0.0002 | *t*(8) = 6.696 |
| Fig. 6o | AAV-mCherry (n = 5) | AAV-shRNA (n = 5) | Two-way RM ANOVA  with Bonferroni post hoc analysis | *P* < 0.0001 | *F*(1, 8) = 63.68 |
| Fig. 6u | AAV-mCherry (n = 5) | AAV-shRNA (n = 5) | Two-way RM ANOVA  with Bonferroni post hoc analysis | *P* = 0.0009 | *F*(1, 8) = 26.00 |
| Fig. S1b | Saline (n = 7) | CFA 3D (n = 7) | Two-way RM ANOVA  with Bonferroni post hoc analysis | *P* < 0.0001 | *F*(1,12) =170.0 |
|  | 1D | |  | *P* < 0.0001 |  |
|  | 3D | |  | *P* < 0.0001 |  |
|  | 5D | |  | *P* < 0.0001 |  |
|  | 7D | |  | *P* < 0.0001 |  |
|  | 10D | |  | *P* < 0.0001 |  |
|  | 14D | |  | *P* = 0.2033 |  |
| Fig. S4b | Saline (n = 20) | CFA 3D (n = 20) | Two-way RM ANOVA  with Bonferroni post hoc analysis | *P* = 0.0073 | *F*(1, 38) = 8.025 |
| Fig. S4c | Saline (n = 20) | CFA 3D (n = 20) | Unpaired Student’s t-test | *P* = 0.004 | *t*(38) = 3.069 |
| Fig. S4d | Saline (n = 20) | CFA 3D (n = 20) | Unpaired Student’s t-test | *P* = 0.0157 | *t*(38) = 2.528 |
| Fig. S4e | Saline (n = 20) | CFA 3D (n = 20) | Unpaired Student’s t-test | *P* = 0.4123 | *t*(38) = 0.8290 |
| Fig. S4g | Saline (n = 20) | CFA 3D (n = 26) | Unpaired Student’s t-test | *P* = 0.0093 | *t*(44) = 2.721 |
| Fig. S5c |  |  | One-way RM ANOVA  with Bonferroni post hoc analysis | *P* < 0.0001 | *F*(2, 73) = 11.00 |
|  | Saline-mCherry (n = 23) | CFA 3D-mCherry (n = 25) |  | *P* = 0.0011 |  |
|  | CFA 3D-mCherry (n = 25) | CFA 3D-hM4Di (n = 28) |  | *P* < 0.0001 |  |
| Fig. S5d | CFA 3D-mCherry (n = 7) | CFA 3D-hM4Di (n = 7) | Two-way RM ANOVA  with Bonferroni post hoc analysis | *P* < 0.0001 | *F*(1, 12) = 148.9 |
|  | CFA 3D | |  | *P* > 0.9999 |  |
|  | Post CNO 0.5 h | |  | *P* > 0.9999 |  |
|  | Post CNO 1.0 h | |  | *P* = 0.9986 |  |
|  | Post CNO 1.5 h | |  | *P* = 0.0281 |  |
|  | Post CNO 2.0 h | |  | *P* = 0.0016 |  |
|  | Post CNO 2.5 h | |  | *P* < 0.0001 |  |
|  | Post CNO 3.0 h | |  | *P* = 0.0005 |  |
|  | Post CNO 3.5 h | |  | *P* = 0.1554 |  |
|  | Post CNO 4.0 h | |  | *P* = 0.7528 |  |
| Fig. S7a | Saline (n = 6) | CFA 3D (n = 6) | Unpaired Student’s t-test | *P* = 0.0053 | *t*(10) = 3.549 |
| Fig. S7b | Saline (n = 5) | CFA 3D (n = 5) | Unpaired Student’s t-test | *P* = 0.0190 | *t*(8) = 2.930 |
| Fig. S7c | Saline (n = 5) | CFA 3D (n = 5) | Unpaired Student’s t-test | *P* = 0.4597 | *t*(8) = 0.7767 |
| Fig. S8c | Saline (n = 20) | CFA 3D (n = 20) | Two-way RM ANOVA  with Bonferroni post hoc analysis | *P* = 0.0001 | *F*(1, 38) = 18.63 |
| Fig. S9b | Saline (n = 20) | CFA 3D (n = 20) | Two-way RM ANOVA  with Bonferroni post hoc analysis | *P* = 0.0001 | *F*(1, 38) = 18.63 |
| Fig. S9c | Saline (n = 20) | CFA 3D (n = 20) | Unpaired Student’s t-test | *P* < 0.0001 | *t*(38) = 6.325 |
| Fig. S9d | Saline (n = 20) | CFA 3D (n = 20) | Two-way RM ANOVA  with Bonferroni post hoc analysis | *P* < 0.0001 | *F*(1, 38) = 24.62 |
| Fig. S9f | Saline (n = 18) | CFA 3D (n = 18) | Two-way RM ANOVA  with Bonferroni post hoc analysis | *P* = 0.0287 | *F*(1, 34) = 5.218 |
| Fig. S9i | CFA 3D-ACSF (n = 13) | CFA 3D-ZD7288 (n = 13) | Two-way RM ANOVA  with Bonferroni post hoc analysis | *P* = 0.0141 | *F*(1, 24) = 7.017 |
| Fig. S9j | CFA 3D-ACSF (n = 13) | CFA 3D-ZD7288 (n = 13) | Unpaired Student’s t-test | *P* = 0.0168 | *t*(24) = 2.571 |
| Fig. S9l | CFA 3D-ACSF (n = 13) | CFA 3D-ZD7288 (n = 13) | Two-way RM ANOVA  with Bonferroni post hoc analysis | *P* < 0.0001 | *F*(1, 24) = 29.41 |
| Fig. S9m | CFA 3D-ACSF (n = 13) | CFA 3D-ZD7288 (n = 13) | Unpaired Student’s t-test | *P* = 0.0001 | *t*(24) = 4.607 |
| Fig. S9n | CFA 3D-ACSF (n = 13) | CFA 3D-ZD7288 (n = 13) | Two-way RM ANOVA  with Bonferroni post hoc analysis | *P* < 0.0001 | *F*(1, 24) = 36.66 |
| Fig. S9o | CFA 3D-ACSF (n = 13) | CFA 3D-ZD7288 (n = 13) | Unpaired Student’s t-test | *P* < 0.0001 | *t*(24) = 5.400 |
| Fig. S9p | CFA 3D-ACSF (n = 13) | CFA 3D-ZD7288 (n = 13) | Unpaired Student’s t-test | *P* = 0.0067 | *t*(24) = 2.970 |
| Fig. S9q | ACSF vs ZD7288 | |  |  |  |
|  | Saline-ACSF (n = 7) | Saline-ZD7288 (n = 7) | Two-way RM ANOVA  with Bonferroni post hoc analysis | *P* = 0.2107 | *F*(1, 12) = 1.749 |
|  | Post ZD7288 0.5 h | |  | *P* > 0.9999 |  |
|  | Post ZD7288 1.0 h | |  | *P* > 0.9999 |  |
|  | Post ZD7288 1.5 h | |  | *P* = 0.8686 |  |
|  | Post ZD7288 2.0 h | |  | *P* > 0.9999 |  |
|  | Post ZD7288 2.5 h | |  | *P* > 0.9999 |  |
|  | Post ZD7288 3.0 h | |  | *P* > 0.9999 |  |
|  | Post ZD7288 3.5 h | |  | *P* > 0.9999 |  |
|  | Post ZD7288 4.0 h | |  | *P* > 0.9999 |  |
|  | CFA 3D-ACSF (n = 7) | CFA 3D-ZD7288 (n = 7) | Two-way RM ANOVA  with Bonferroni post hoc analysis | *P* < 0.0001 | *F*(1, 8) = 150.2 |
|  | Post ZD7288 0.5 h | | *P* > 0.9999 |  |  |
|  | Post ZD7288 1.0 h | | *P* < 0.0001 |  |  |
|  | Post ZD7288 1.5 h | | *P* < 0.0001 |  |  |
|  | Post ZD7288 2.0 h | | *P* < 0.0001 |  |  |
|  | Post ZD7288 2.5 h | | *P* = 0.0907 |  |  |
|  | Post ZD7288 3.0 h | | *P* > 0.9999 |  |  |
|  | Post ZD7288 3.5 h | | *P* > 0.9999 |  |  |
|  | Post ZD7288 4.0 h | | *P* > 0.9999 |  |  |
| Fig. S12c | Saline (n = 10) | CFA 3D (n = 10) | Unpaired Student’s t-test | *P* = 0.0001 | *t*(18) = 4.964 |
| Fig. S12f | Saline (n = 21) | CFA 3D (n = 37) | Unpaired Student’s t-test | *P* = 0.0009 | *t*(56) = 3.508 |
| Fig. S12o | Saline (n = 128) | CFA 3D (n = 131) | Unpaired Student’s t-test | *P* < 0.0001 | *t*(257) = 6.640 |
| Fig. S12p | Saline (n = 128) | CFA 3D (n = 131) | Unpaired Student’s t-test | *P* < 0.0001 | *t*(257) = 4.444 |
| Fig. S13c |  |  | One-way RM ANOVA  with Bonferroni post hoc analysis | *P* < 0.0001 | *F*(2, 15) = 18.14 |
|  | Sham-mCherry (n = 6) | SNI 7D-mCherry (n = 6) |  | *P* = 0.0003 |  |
|  | SNI 7D-mCherry (n = 6) | SNI 7D-hM4Di (n = 6) |  | *P* = 0.0003 |  |
| Fig. S13f |  |  | One-way RM ANOVA  with Bonferroni post hoc analysis | *P* = 0.0091 | *F*(2, 15) = 6.536 |
|  | Saline-mCherry (n = 6) | CFA 3D-mCherry (n = 6) |  | *P* = 0.0194 |  |
|  | CFA 3D-mCherry (n = 6) | CFA 3D-hM4Di (n = 6) |  | *P* = 0.0219 |  |
| Fig. S13i |  |  | One-way RM ANOVA  with Bonferroni post hoc analysis | *P* = 0.0002 | *F*(2, 69) = 9.937 |
|  | Saline-mCherry (n = 23) | CFA 3D-mCherry (n = 28) |  | *P* = 0.0024 |  |
|  | CFA 3D-mCherry (n = 28) | CFA 3D-hM4Di (n = 21) |  | *P* = 0.0005 |  |
| Fig. S13j | EYFP (n = 5) | eNpHR 3.0-EYFP (n = 7) | Two-way RM ANOVA  with Bonferroni post hoc analysis | *P* < 0.0001 | *F*(1, 10) = 68.00 |
|  | Light on | | *P* < 0.0001 |  |  |
| Fig. S14c | mCherry (n = 5) | ChR2-mCherry (n = 5) | Two-way RM ANOVA  with Bonferroni post hoc analysis | *P* = 0.0040 | *F*(1, 8) = 15.87 |
|  | Light on | |  | *P* = 0.0102 |  |
| Fig. S16c |  |  | One-way RM ANOVA  with Bonferroni post hoc analysis | *P* < 0.0001 | *F*(2, 15) = 78.76 |
|  | Saline-ACSF (n = 6) | CFA 3D-ACSF (n = 6) |  | *P* < 0.0001 |  |
|  | CFA 3D-ACSF (n = 6) | CFA 3D-ZD7288 (n = 6) |  | *P* < 0.0001 |  |
| Fig. S16e | CFA 3D-AAV-mCherry  (n = 5) | CFA 3D-AAV-shRNA  (n = 5) | Two-way RM ANOVA  with Bonferroni post hoc analysis | *P* < 0.0001 | *F*(1, 8) = 64.15 |
| Fig. S16i | CFA 3D-AAV-mCherry  (n = 5) | CFA 3D-AAV-shRNA  (n = 5) | Two-way RM ANOVA  with Bonferroni post hoc analysis | *P* = 0 .0004 | *F*(1, 8) = 33.37 |
| Fig. S17e | AAV-control (n = 5) | AAV-HCN2 (n = 5) | Unpaired Student’s t-test | *P* = 0.0014 | *t*(8) = 4.790 |
| Fig. S17g | AAV-control (n = 14) | AAV-HCN2 (n = 15) | Two-way RM ANOVA  with Bonferroni post hoc analysis | *P* < 0.0001 | *F*(1, 27) = 26.06 |
| Fig. S17i | AAV-control (n = 17) | AAV-HCN2 (n = 17) | Two-way RM ANOVA  with Bonferroni post hoc analysis | *P* < 0.0001 | *F*(1, 32) = 24.68 |
| Fig. S17j | AAV-control (n = 17) | AAV-HCN2 (n = 17) | Unpaired Student’s t-test | *P* = 0.8595 | *t*(32) = 0.1785 |
| Fig. S17l | AAV-control (n = 17) | AAV-HCN2 (n = 17) | Two-way RM ANOVA  with Bonferroni post hoc analysis | *P* = 0.0006 | *F*(1, 32) = 14.35 |
| Fig. S17m | AAV-control (n = 17) | AAV-HCN2 (n = 17) | Unpaired Student’s t-test | *P* = 0.6704 | *t*(32) = 0.4295 |
| Fig. S17n | AAV-control (n = 17) | AAV-HCN2 (n = 17) | Two-way RM ANOVA  with Bonferroni post hoc analysis | *P* < 0.0001 | *F*(1, 32) = 35.93 |
| Fig. S17o | AAV-control (n = 17) | AAV-HCN2 (n = 17) | Unpaired Student’s t-test | *P* = 0.0068 | *t*(32) = 2.892 |
| Fig. S17p | AAV-control (n = 17) | AAV-HCN2 (n = 17) | Unpaired Student’s t-test | *P* = 0.0002 | *t*(32) = 4.281 |
| Fig. S17v | AAV-control (n = 7) | AAV-HCN2 (n = 7) | Two-way RM ANOVA  with Bonferroni post hoc analysis | *P* < 0.0001 | *F*(1,12) =36.82 |
| Fig. S17y | AAV-HCN2-EYFP  (n = 7) | AAV-HCN2-eNpHR 3.0  (n = 7) | Two-way RM ANOVA  with Bonferroni post hoc analysis | *P* < 0.0166 | *F*(1, 10) = 8.242 |
|  | Light on |  | P < 0.0001 |  |  |
| Fig. S18a | Sham (n = 8) | SNI 7D (n = 8) | Unpaired Student’s t-test | *P* = 0.0154 | *t*(14) = 2.759 |
| Fig. S18d | SNI 7D-ACSF (n = 10) | SNI 7D-SQ22536 (n = 10) | Two-way RM ANOVA  with Bonferroni post hoc analysis | *P* = 0.0218 | *F*(1, 18) = 6.309 |
| Fig. S18f | SNI 7D-ACSF (n = 15) | SNI 7D-SQ22536 (n = 10) | Two-way RM ANOVA  with Bonferroni post hoc analysis | *P* = 0.0003 | *F*(1, 23) = 17.64 |
| Fig. S18g | SNI 7D-ACSF (n = 15) | SNI 7D-SQ22536 (n = 10) | Unpaired Student’s t-test | *P* = 0.0006 | *t*(23) = 3.976 |
| Fig. S18i | SNI 7D-ACSF (n = 15) | SNI 7D-SQ22536 (n = 10) | Two-way RM ANOVA  with Bonferroni post hoc analysis | *P* < 0.0001 | *F*(1, 23) = 46.27 |
| Fig. S18j | SNI 7D-ACSF (n = 15) | SNI 7D-SQ22536 (n = 10) | Unpaired Student’s t-test | *P* < 0.0001 | *t*(23) = 5.663 |
| Fig. S18k | SNI 7D-ACSF (n = 15) | SNI 7D-SQ22536 (n = 10) | Two-way RM ANOVA  with Bonferroni post hoc analysis | *P* < 0.0001 | *F*(1, 23) = 46.87 |
| Fig. S18l | SNI 7D-ACSF (n = 15) | SNI 7D-SQ22536 (n = 10) | Unpaired Student’s t-test | *P* = 0.0004 | *t*(23) = 4.180 |
| Fig. S18m | SNI 7D-ACSF (n = 15) | SNI 7D-SQ22536 (n = 10) | Unpaired Student’s t-test | *P* = 0.3392 | *t*(23) = 0.9759 |
| Fig. S18n | SNI 7D-ACSF (n = 7) | SNI 7D-SQ22536 (n = 7) | Two-way RM ANOVA  with Bonferroni post hoc analysis | *P* < 0.0001 | *F*(1, 12) = 76.28 |
|  | SNI 7D |  |  | *P* > 0.9999 |  |
|  | Post SQ22536 0.5 h |  |  | *P* > 0.9999 |  |
|  | Post SQ22536 1.0 h |  |  | *P* = 0.0011 |  |
|  | Post SQ22536 1.5 h |  |  | *P* = 0.0018 |  |
|  | Post SQ22536 2.0 h |  |  | *P* = 0.0143 |  |
|  | Post SQ22536 2.5 h |  |  | *P* = 0.0231 |  |
|  | Post SQ22536 3.0 h |  |  | *P* = 0.9308 |  |
| Fig. S19a | Saline (n = 9) | CFA 3D (n = 9) | Unpaired Student’s t-test | *P* = 0.0303 | *t*(16) = 2.377 |
| Fig. S19d | CFA 3D-ACSF (n = 10) | CFA 3D-SQ22536 (n = 10) | Two-way RM ANOVA  with Bonferroni post hoc analysis | *P* < 0.0001 | *F*(1, 18) = 42.96 |
| Fig. S19f | CFA 3D-ACSF (n = 12) | CFA 3D-SQ22536 (n = 10) | Two-way RM ANOVA  with Bonferroni post hoc analysis | *P* = 0.0032 | *F*(1, 20) = 11.25 |
| Fig. S19g | CFA 3D-ACSF (n = 12) | CFA 3D-SQ22536 (n = 10) | Unpaired Student’s t-test | *P* < 0.0001 | *t*(20) = 5.769 |
| Fig. S19i | CFA 3D-ACSF (n = 12) | CFA 3D-SQ22536 (n = 10) | Two-way RM ANOVA  with Bonferroni post hoc analysis | *P* = 0.0001 | *F*(1, 20) = 22.91 |
| Fig. S19j | CFA 3D-ACSF (n = 12) | CFA 3D-SQ22536 (n = 10) | Unpaired Student’s t-test | *P* < 0.0001 | *t*(20) = 7.449 |
| Fig. S19k | CFA 3D-ACSF (n = 12) | CFA 3D-SQ22536 (n = 10) | Two-way RM ANOVA  with Bonferroni post hoc analysis | *P* < 0.0001 | *F*(1, 20) = 43.58 |
| Fig. S19l | CFA 3D-ACSF (n = 12) | CFA 3D-SQ22536 (n = 10) | Unpaired Student’s t-test | *P* = 0.0155 | *t*(20) = 2.645 |
| Fig.S19m | CFA 3D-ACSF (n = 12) | CFA 3D-SQ22536 (n = 10) | Unpaired Student’s t-test | *P* = 0.6190 | *t*(20) = 0.5051 |
| Fig. S19n | CFA 3D-ACSF (n = 6) | CFA 3D-SQ22536 (n = 6) | Two-way RM ANOVA  with Bonferroni post hoc analysis | *P* < 0.0001 | *F*(1, 10) = 215.9 |
|  | CFA 3D |  | *P* > 0.9999 |  |  |
|  | Post SQ22536 0.5 h |  | *P* > 0.9999 |  |  |
|  | Post SQ22536 1.0 h |  | *P* < 0.0001 |  |  |
|  | Post SQ22536 1.5 h |  | *P* < 0.0001 |  |  |
|  | Post SQ22536 2.0 h |  | *P* < 0.0001 |  |  |
|  | Post SQ22536 2.5 h |  | *P* = 0.0251 |  |  |
|  | Post SQ22536 3.0 h |  | *P* > 0.9999 |  |  |

**Table S2 KEY RESOURCES TABLE**

| **Resource or Reagent** | **Source** | **Identifier** |
| --- | --- | --- |
| **Racterial and Virus Strains** | | |
| rAAV-CaMKIIa-GCaMp6m-WPRE-hGH pA | BrainVTA | Cat#PT-0111 |
| rAAV-CaMKIIa-GCaMp6f-WPRE-hGH pA | BrainVTA | Cat#PT-0119 |
| rAAV-EF1α-DIO-ΔRVG-WPRE-hGH pA | BrainVTA | Cat#PT-0023 |
| rAAV-EF1α-DIO-H2B-EGFP-T2A-TVA-WPRE-hGH pA | BrainVTA | Cat#PT-0021 |
| RV-ENVA-ΔG-dsRed | BrainVTA | Cat#R01002 |
| rAAV-CaMKII-hChR2(H134R)-mCherry-WPRE-hGH pA | BrainVTA | Cat#PT-0297 |
| rAAV-CaMKII-eNpHR3.0-EYFP-WPRE-hGH pA | BrainVTA | Cat#PT-0008 |
| rAAV-CaMKII-EYFP-WPRE- hGH pA | BrainVTA | Cat#PT-0107 |
| rAAV-CaMKIIa-hM4D(Gi)-mCherry-WPRE-hGH pA | BrainVTA | Cat#PT-0050 |
| rAAV-CaMKIIa-mCherry-WPRE-hGH pA | BrainVTA | Cat#PT-0108 |
| rAAV-VGLUT2-EGFP-WPRE-hGH-pA | BrainVTA | Cat#PT-1886 |
| rAAV-Ef1α-DIO-EYFP-WPRE-pA | BrainVTA | Cat#PT-0012 |
| rAAV-hSyn-Cre-EGFP-WPRE-hGH pA | BrainVTA | Cat#PT-1168 |
| rAAV-CMV-DIO-(mCherry-U6)-shRNA(HCN2)-WPRE-pA | BrainVTA | Cat#PT-3962 |
| rAAV-CMV-DIO-(mCherry-U6)-shRNA(scramble)-WPRE-pA | BrainVTA | Cat#PT-2788 |
| rAAV-CMV-DIO-HCN2-3xflag-WPRE-PA | BrainVTA | Cat#PT-6172 |
| rAAV-CMV-DIO-HCN2-3xflag-WPRE-PA | BrainVTA | Cat#PT-1390 |
| **Antibodies** | | |
| Rabbit anti-glutamate | Sigma | Cat#G6642 |
| Rabbit anti-c-Fos | SYSY | Cat#226003 |
| Mouse anti-glutamate | Sigma | Cat#G9282 |
| Mouse beta-actin | Absin | Cat#abs137975 |
| Rabbit anti-HCN2 | Alomone | Cat#APC-030 |
| Rabbit anti-HCN3 | Alomone | Cat#APC-057 |
| Rabbit anti-HCN4 | Alomone | Cat#APC-052 |
| Rabbit anti-flag tag | Proteintech | Cat#20543-1-AP |
| Donkey anti-rabbit IgG Alexa 488 | Invitrogen | Cat#A21206 |
| Donkey anti-mouse IgG Alexa 594 | Invitrogen | Cat#A21203 |
| Donkey anti-rabbit IgG Alexa 594 | Invitrogen | Cat#A21207 |
| Donkey anti-rabbit IgG Alexa 647 | Invitrogen | Cat#A31573 |
| **Chemicals, Peptides, and Recombinant Proteins** | | |
| ZD7288 | Sigma | Cat#z3777 |
| SQ22536 | MCE | Cat#HY-100396 |
| cAMP ELISA Kit | Sangon Biotech | Cat# D770001-0096 |
| Neuronbictin-488 | Invitrogen | Cat#SP-1125 |
| DAPI | Sigma | Cat#D9542 |
| Clozapine-N-Oxide (CNO) | Sigma | Cat#C0832 |
| Picrotoxin (PTX) | Sigma | Cat#R284556 |
| Tetrodotoxin (TTX) | Tocris Bioscience | Cat#1069 |
| 6,7-dinitroquinoxaline-2,3-dione (DNQX) | Sigma | Cat#2379-57-9 |
| CsCl | Sigma | Cat#7647-17-8 |
| TEA-Cl | Sigma | Cat#56-34-8 |
| 4-AP | Sigma | Cat#20263-07-4 |
| Carprofen | Sigma | Cat#PHR1452 |
| Dexamethasone | MedChemExpress | Cat#HY-14686 |
| Enrofloxacin | MedChemExpress | Cat#HY-B0502 |
| **Experimental models: Organisms/Strains** | | |
| Mouse: C57BL/6J | Charles River | N/A |
| Mouse: CaMKⅡ-ires-Cre | Jackson Laboratories | Cat#005359 |
| Mouse: Ai 14 | Jackson Laboratories | Cat#007914 |
| **Software and Algorithms** | | |
| Illustrator CS6 | Adobe | https://www.adobe.com/products/illustrator.html |
| ZEN | Zeiss | https://www.zeiss.com/microscopy/us/products/  microscope-software/zen-lite.html |
| Graphpad Prism 8.0 | GraphPad software | https://www.graphpad.com/scientific-software/prism/ |
| MatlabR2020b | MathWorks | https://www.mathworks.com/products/new_products/release2020b.html |
| Offline sorter Version 4 | Plexon | https://plexon.com/software-downloads/ |
| Neuroexplorer Version 5 | Plexon | https://plexon.com/software-downloads/ |
| Imagej | National Institutes of Health | https://imagej.net/imagej-wiki-static/fiji |
| Inper Studio | Inper Ltd. | https://www.inper.com/ |
| **Others** | | |
| Optogenetic fibers | Inper | N/A |
| Electrode wire for tetrode | California fine wire | N/A |
