# Supplementary figures and images for: Up-regulation of HCN2 channels in a thalamocortical circuit mediates allodynia in mice
Source: Natl Sci Rev. 2022 Nov 30;10(2):nwac275. doi: 10.1093/nsr/nwac275 (PMC9945406; doi:10.1093/nsr/nwac275)

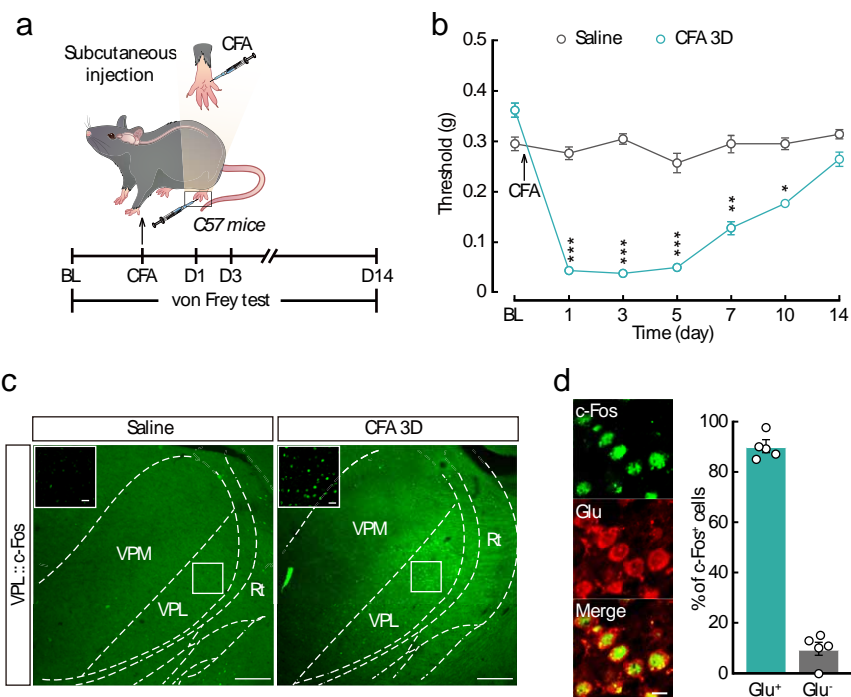

Supplement: nwac275_Supplemental_Files [file nwac275_supplemental_files.zip › Fig. S1.pdf]

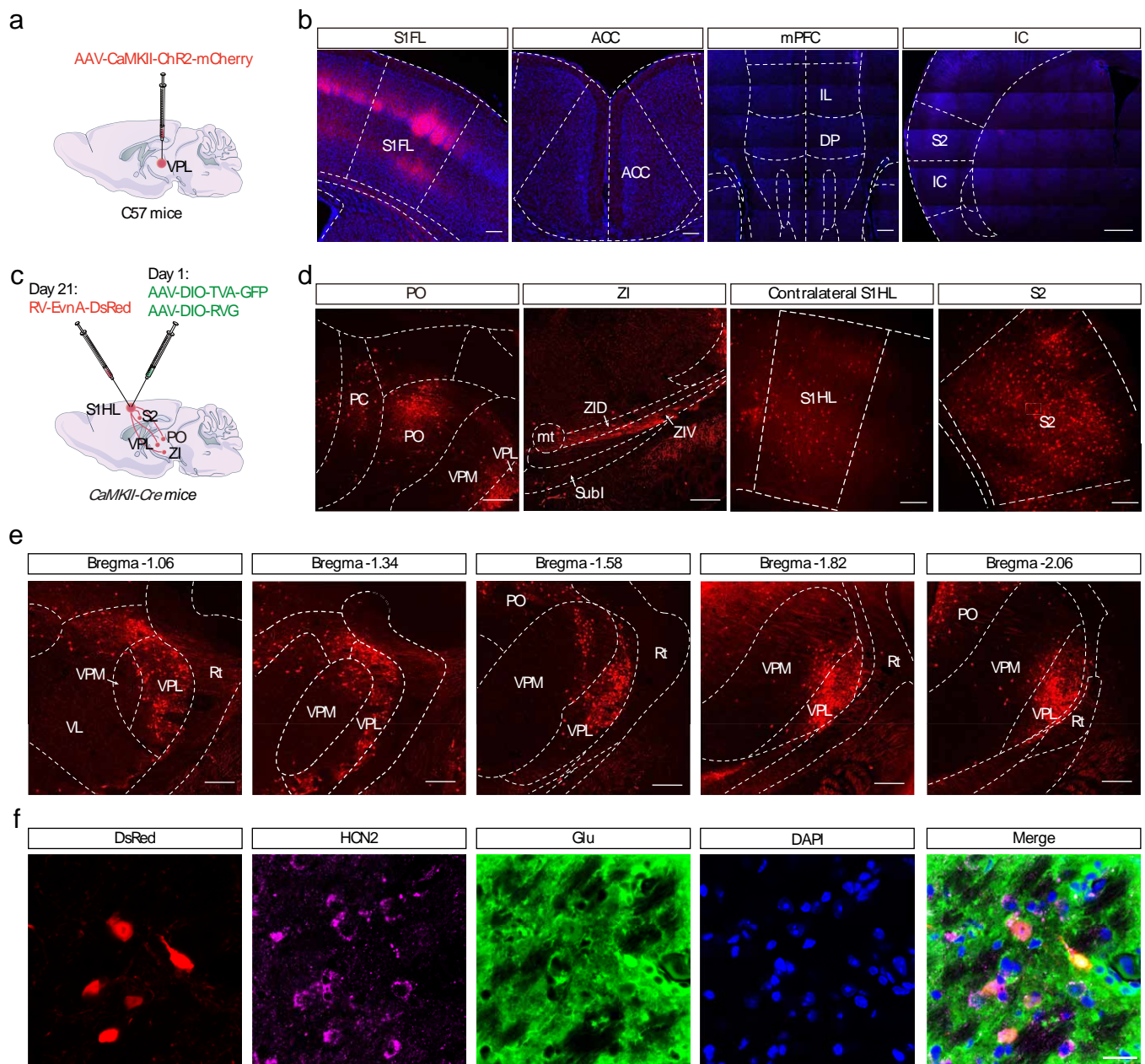

Supplement: nwac275_Supplemental_Files [file nwac275_supplemental_files.zip › Fig. S10.pdf]

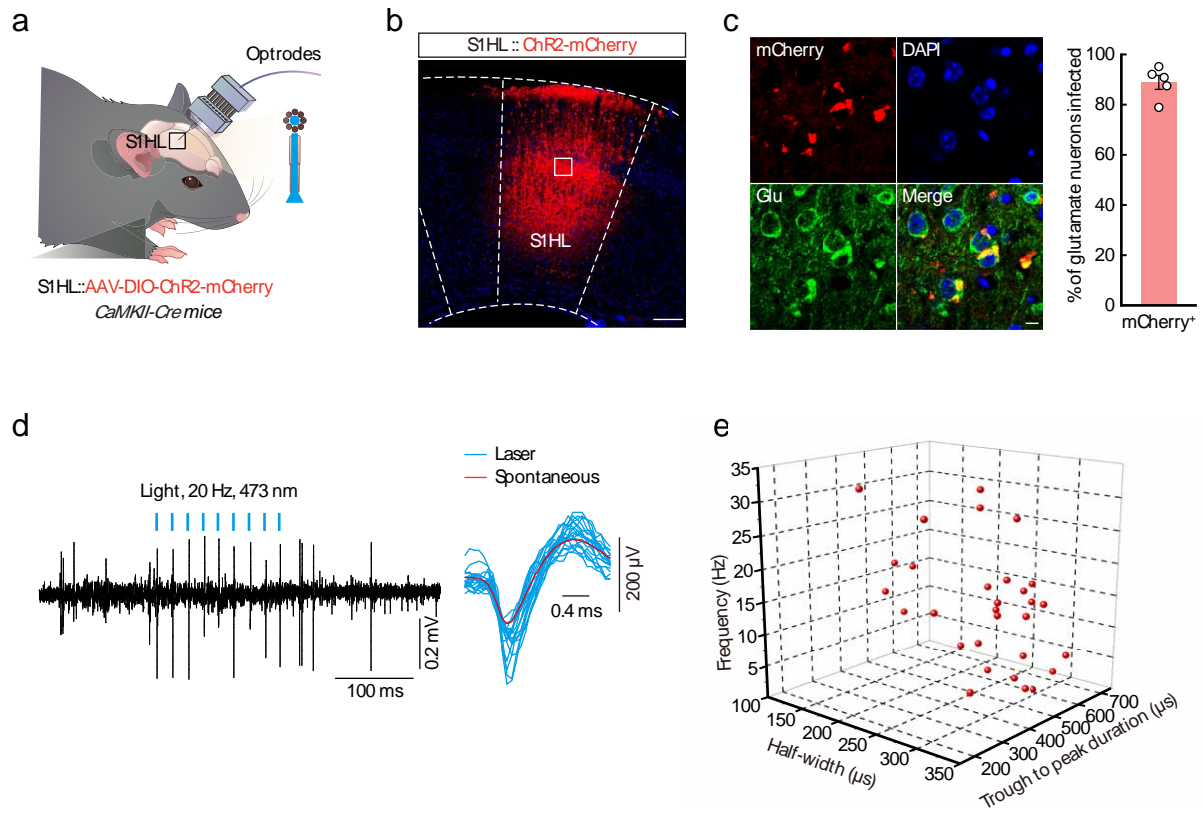

Supplement: nwac275_Supplemental_Files [file nwac275_supplemental_files.zip › Fig. S11.pdf]

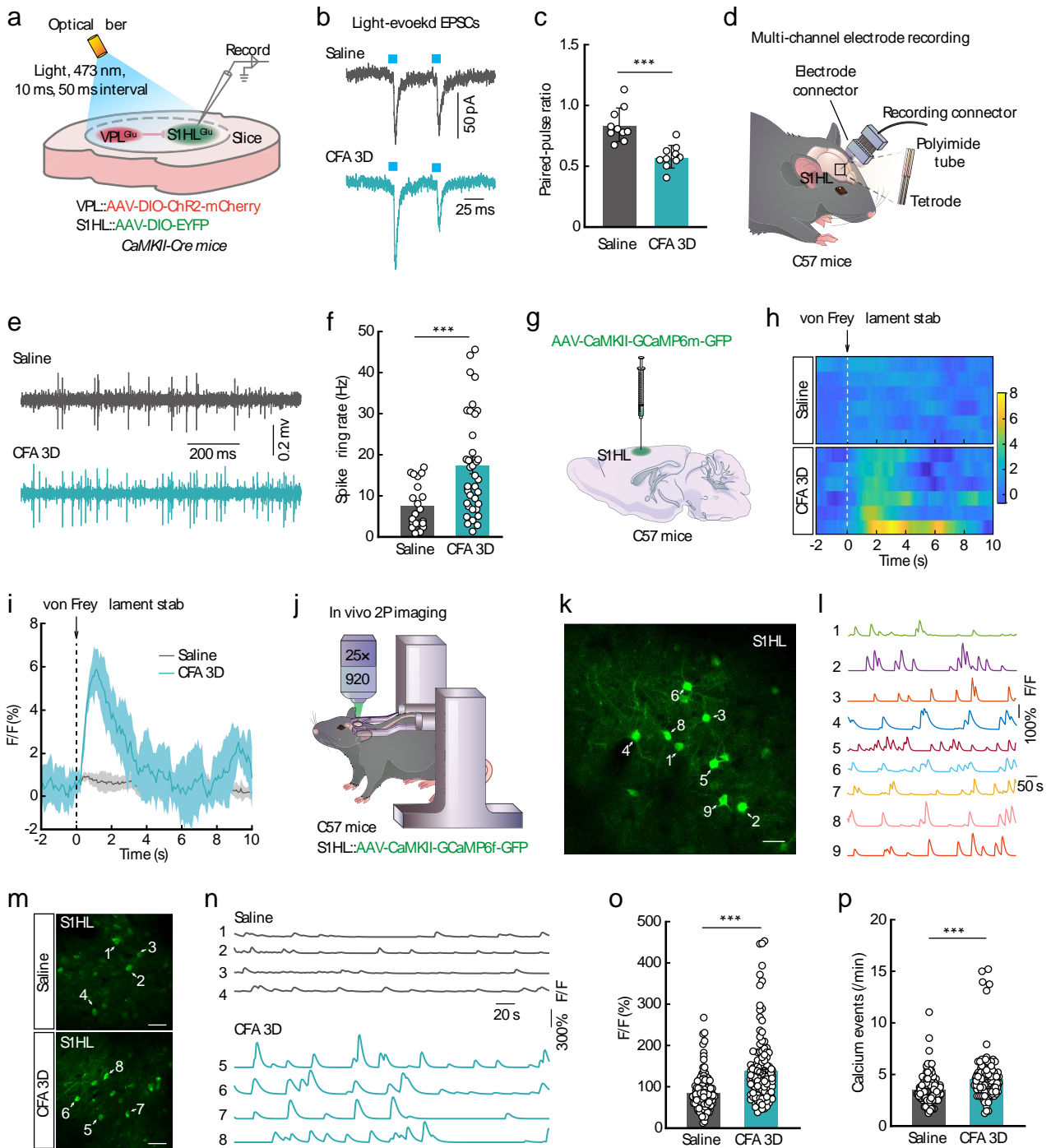

Supplement: nwac275_Supplemental_Files [file nwac275_supplemental_files.zip › Fig. S12.pdf]

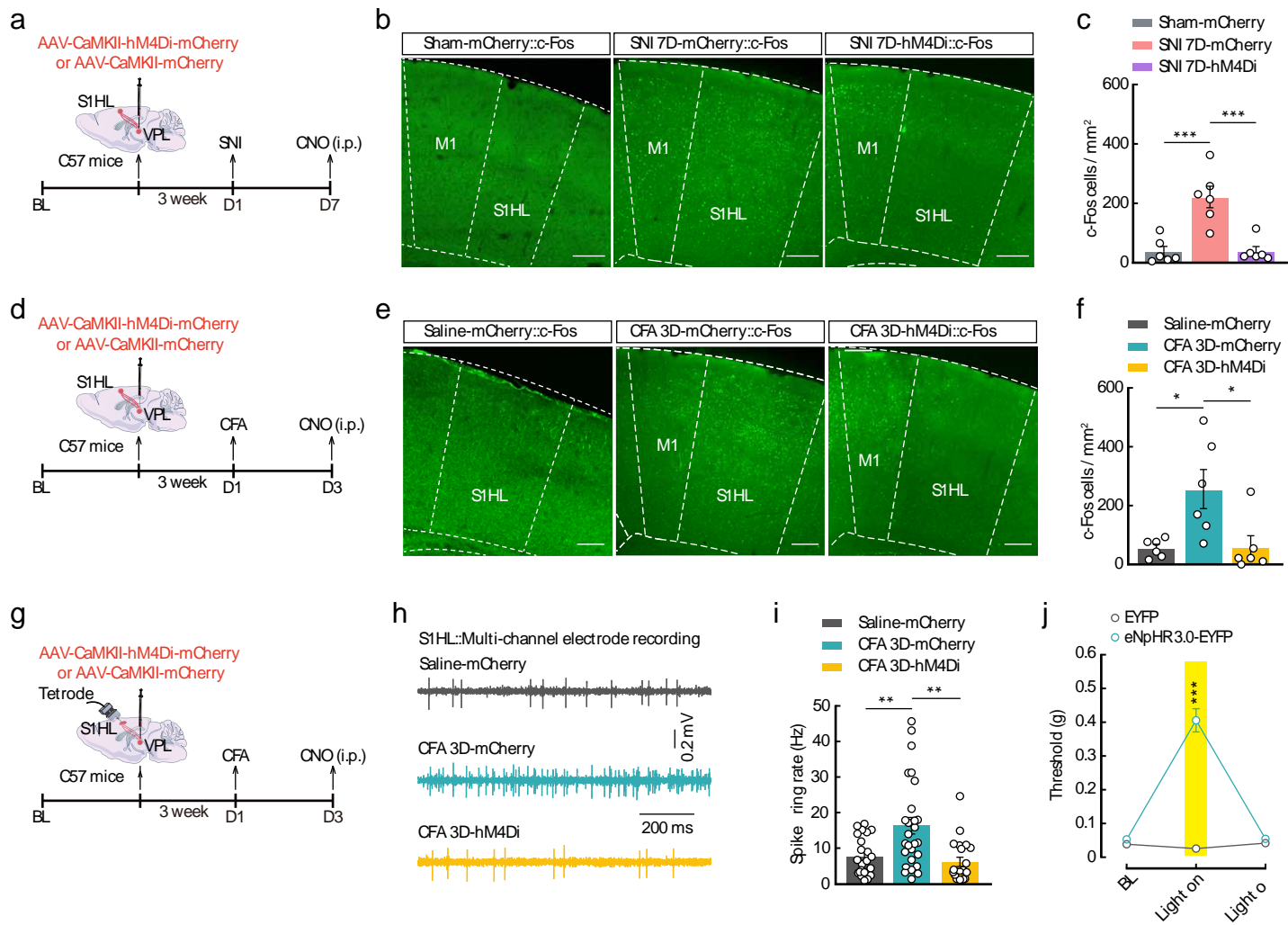

Supplement: nwac275_Supplemental_Files [file nwac275_supplemental_files.zip › Fig. S13.pdf]

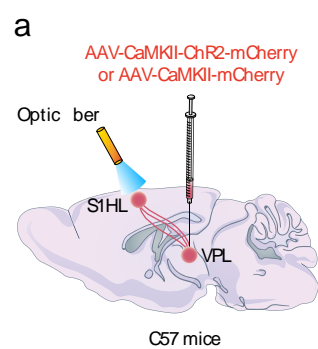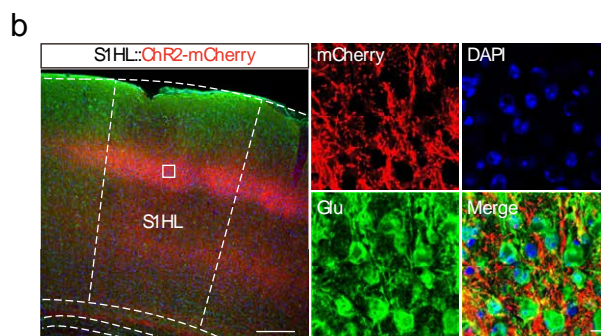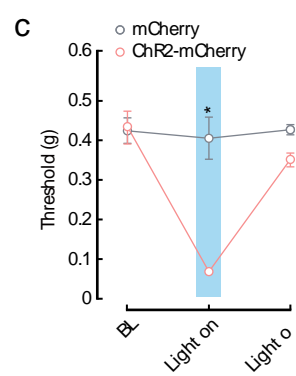

Supplement: nwac275_Supplemental_Files [file nwac275_supplemental_files.zip › Fig. S14.pdf]

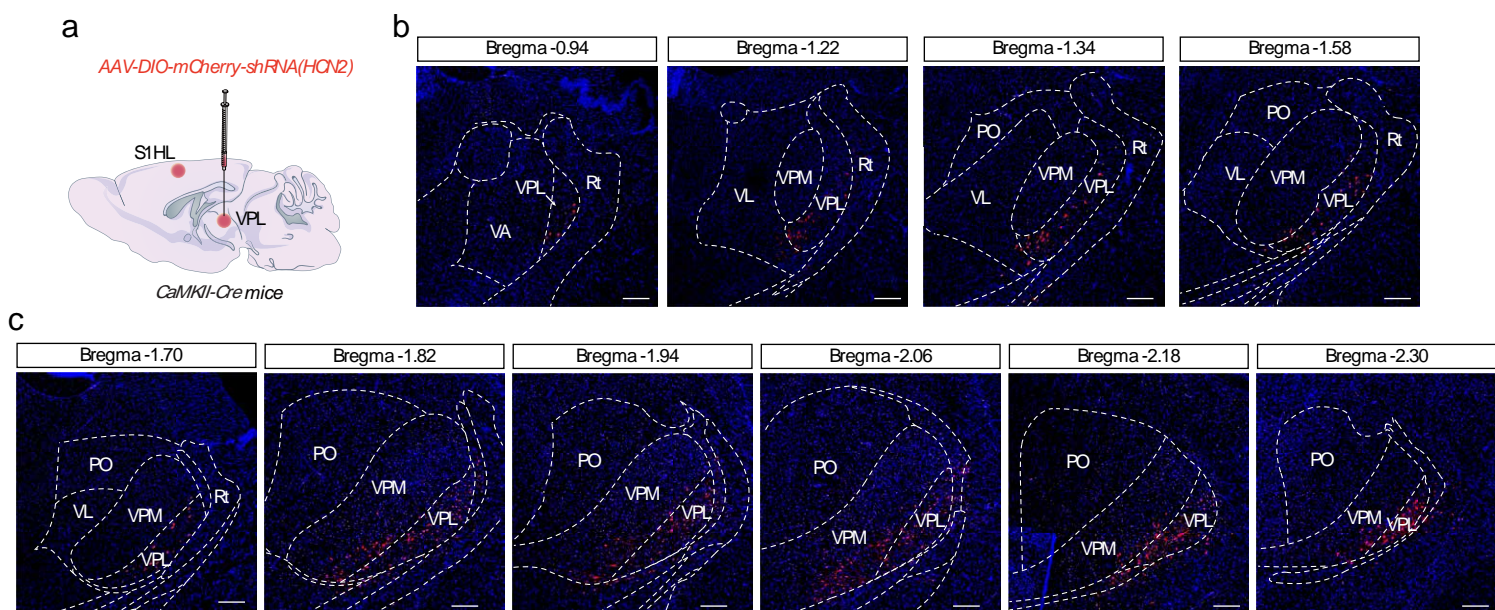

Supplement: nwac275_Supplemental_Files [file nwac275_supplemental_files.zip › Fig. S15.pdf]

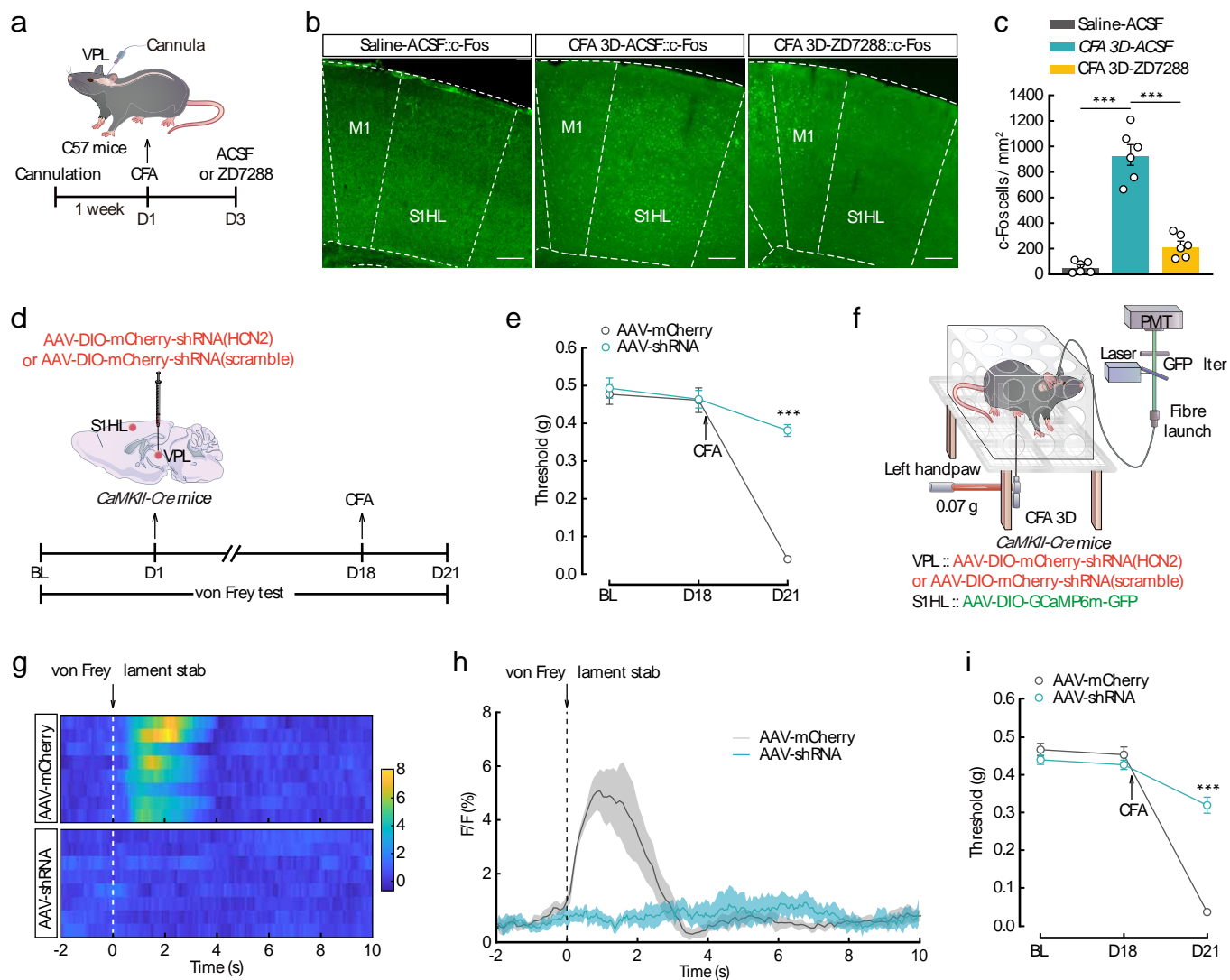

Supplement: nwac275_Supplemental_Files [file nwac275_supplemental_files.zip › Fig. S16.pdf]

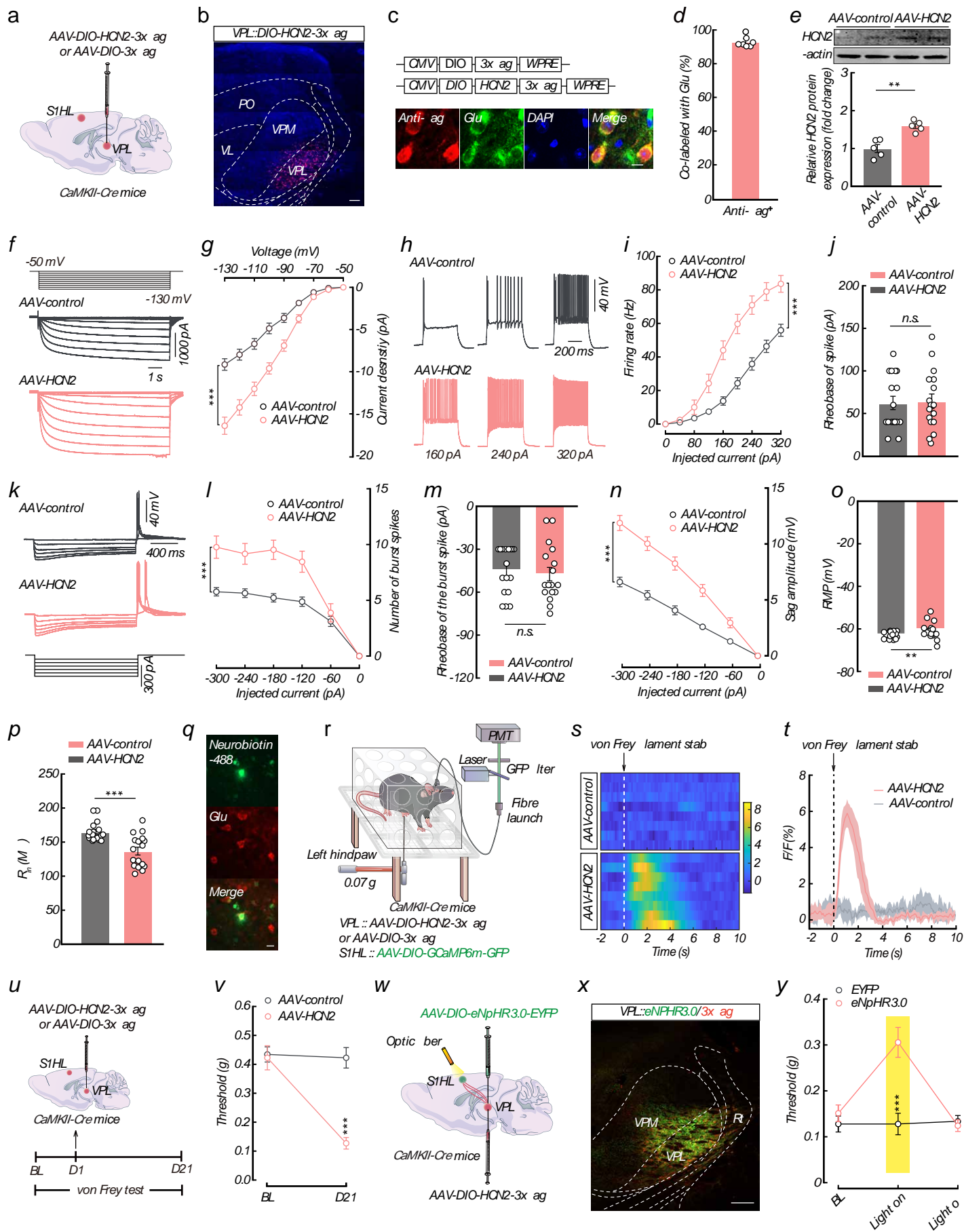

Supplement: nwac275_Supplemental_Files [file nwac275_supplemental_files.zip › Fig. S17.pdf]

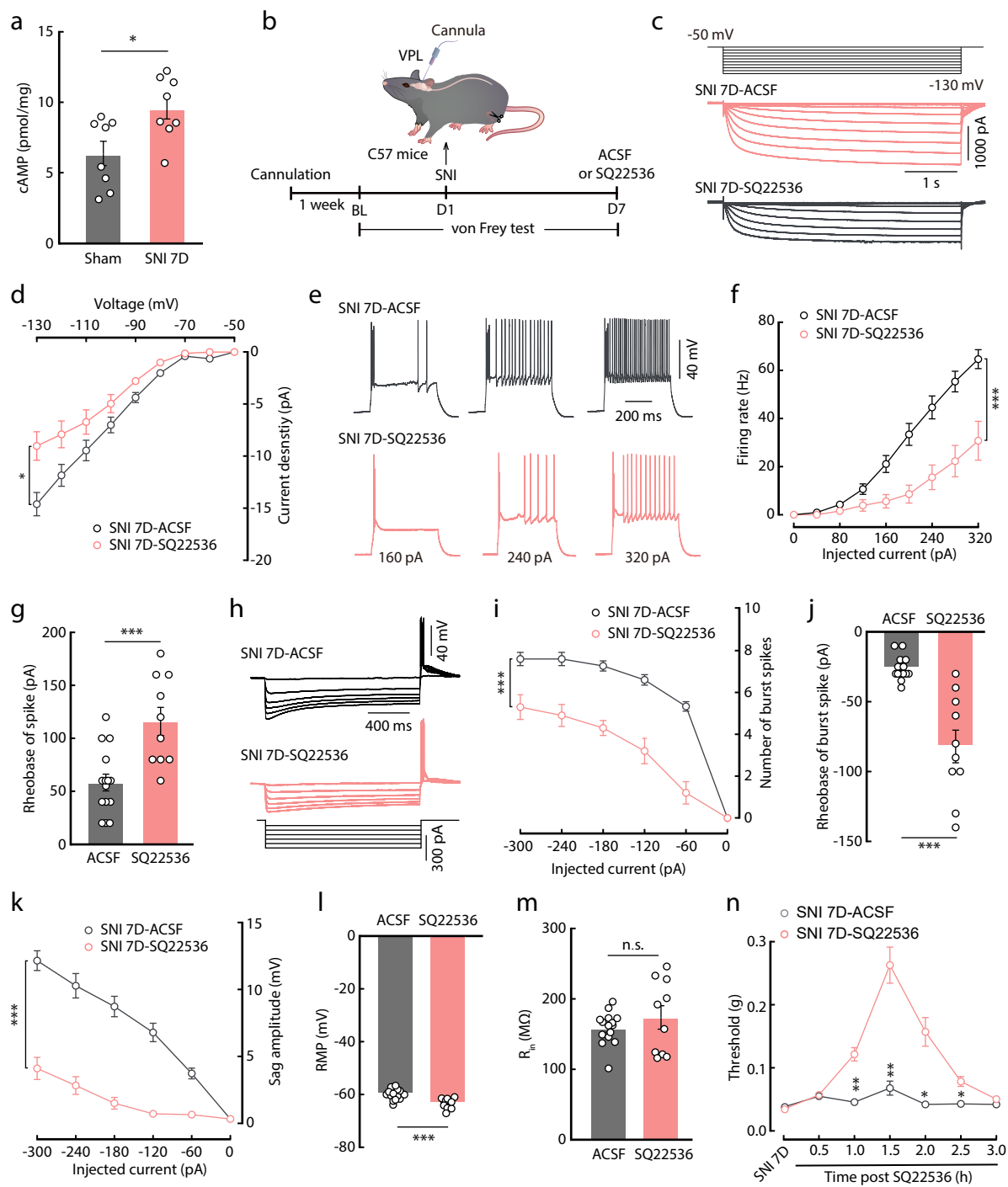

Supplement: nwac275_Supplemental_Files [file nwac275_supplemental_files.zip › Fig. S18.pdf]

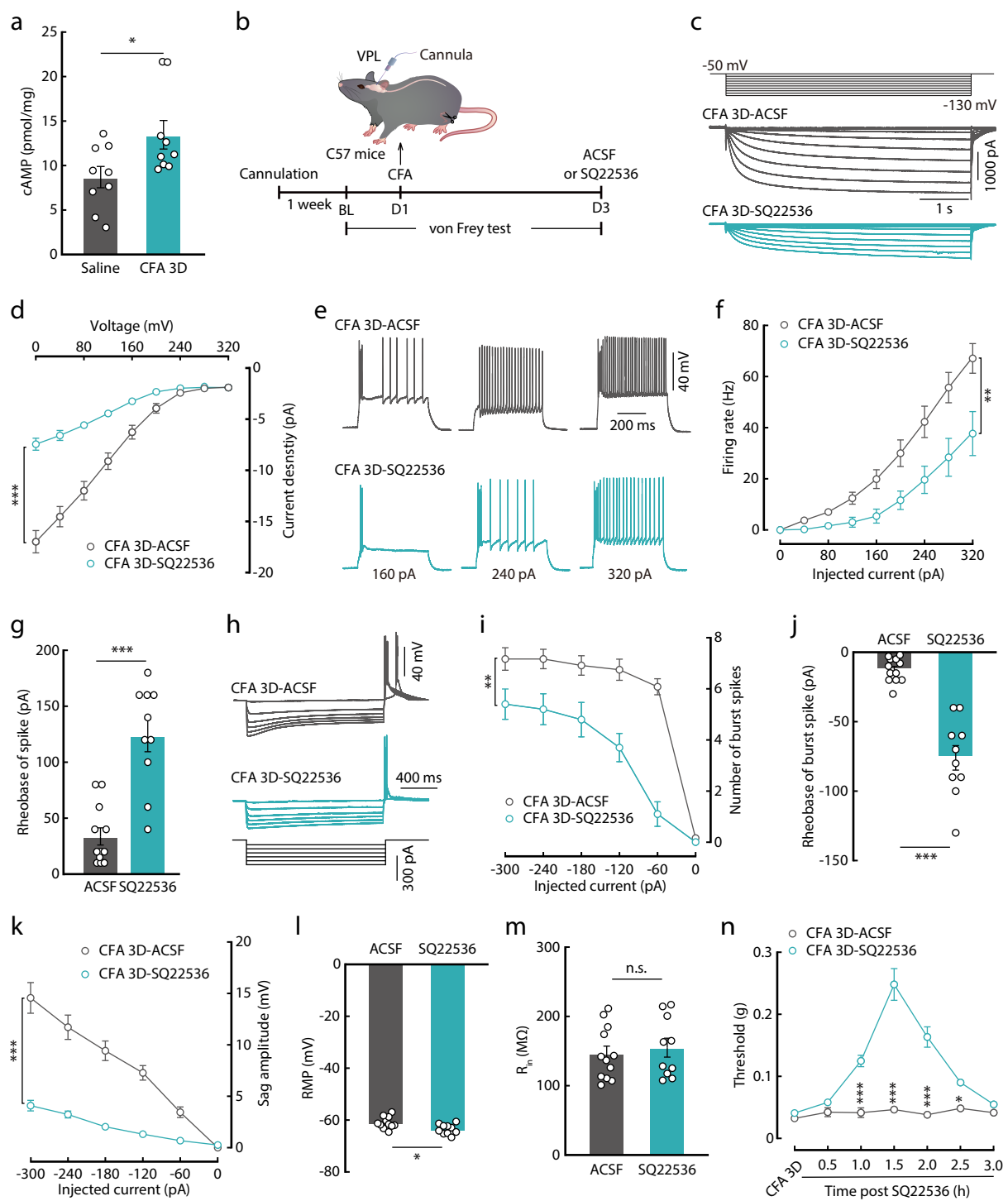

Supplement: nwac275_Supplemental_Files [file nwac275_supplemental_files.zip › Fig. S19.pdf]

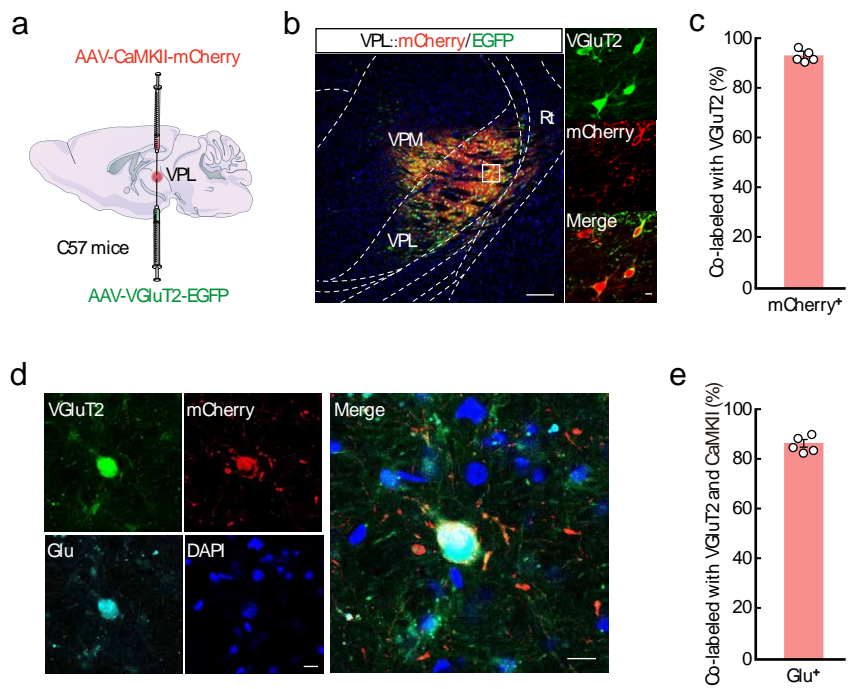

Supplement: nwac275_Supplemental_Files [file nwac275_supplemental_files.zip › Fig. S2.pdf]

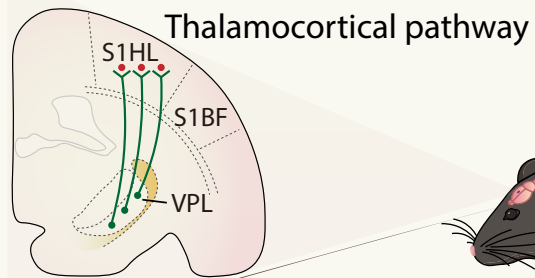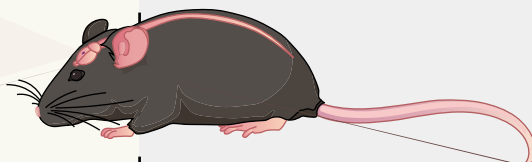

### Control mice

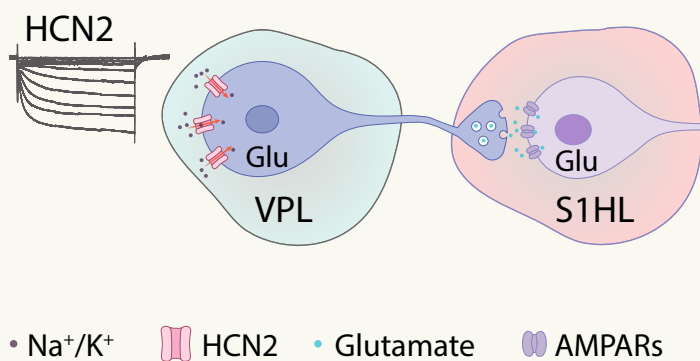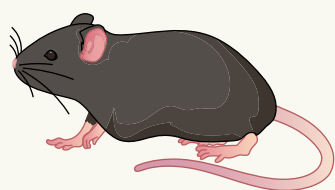

No pain

### Models of chronic pain

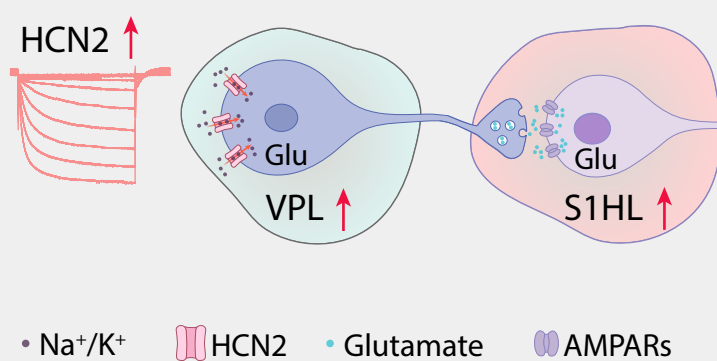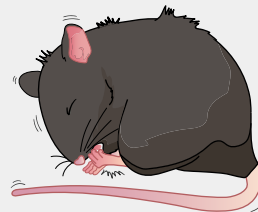

Pain

Knockdown of HCN2

Overexpression of HCN2

Supplement: nwac275_Supplemental_Files [file nwac275_supplemental_files.zip › Fig. S20.pdf]

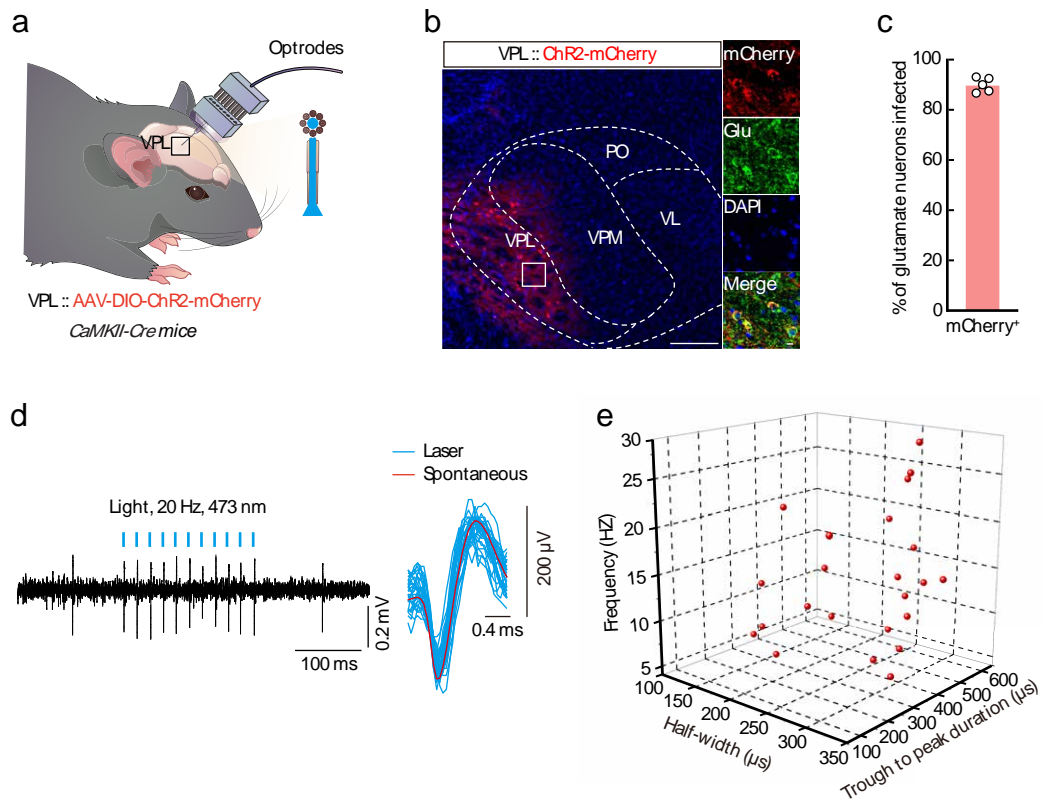

Supplement: nwac275_Supplemental_Files [file nwac275_supplemental_files.zip › Fig. S3.pdf]

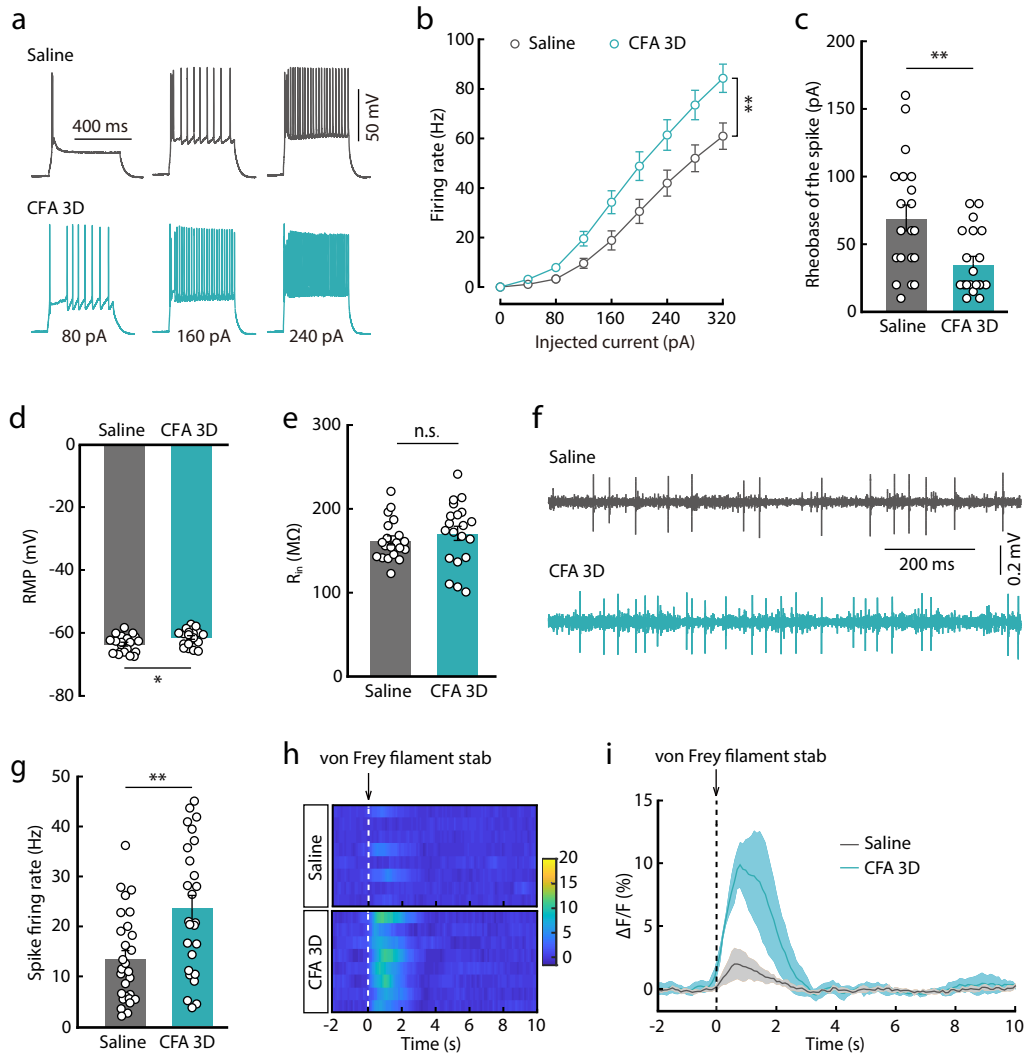

Supplement: nwac275_Supplemental_Files [file nwac275_supplemental_files.zip › Fig. S4.pdf]

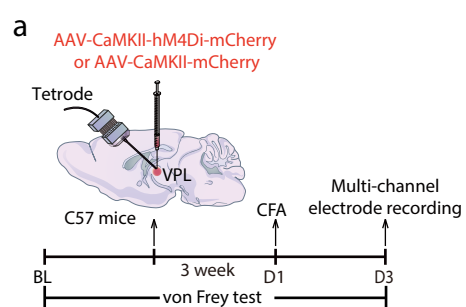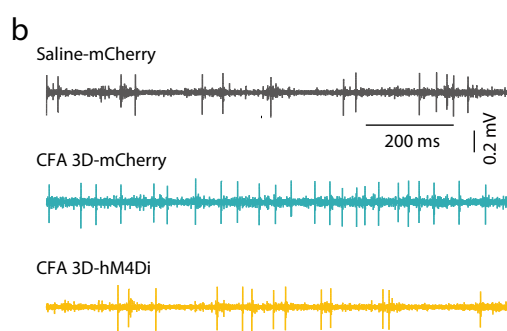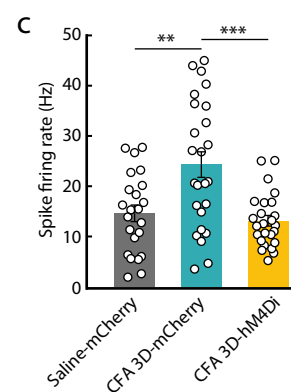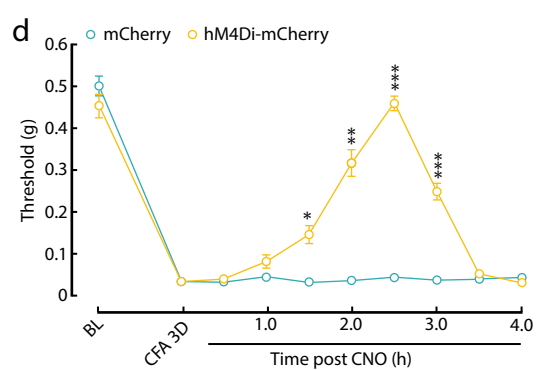

Supplement: nwac275_Supplemental_Files [file nwac275_supplemental_files.zip › Fig. S5.pdf]

a

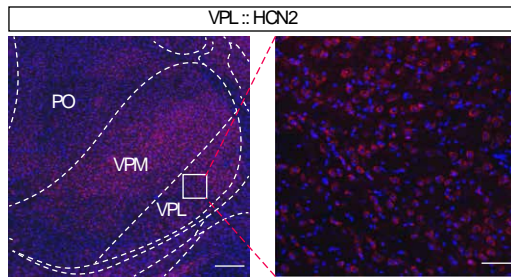

b

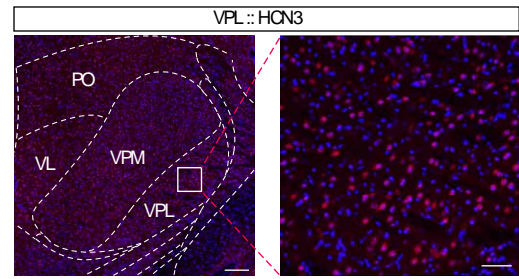

c

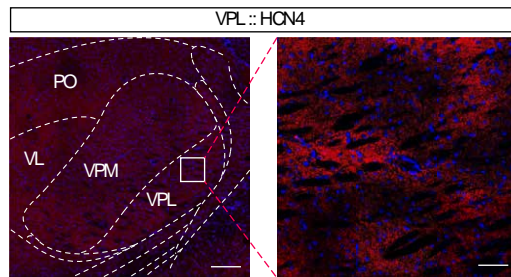

Supplement: nwac275_Supplemental_Files [file nwac275_supplemental_files.zip › Fig. S6.pdf]

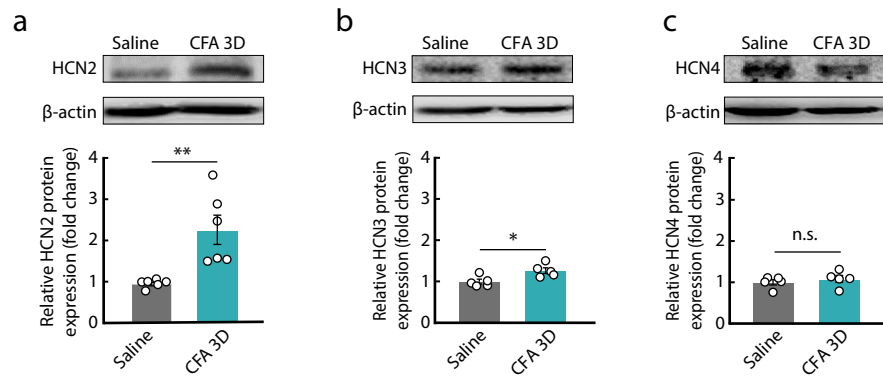

Supplement: nwac275_Supplemental_Files [file nwac275_supplemental_files.zip › Fig. S7.pdf]

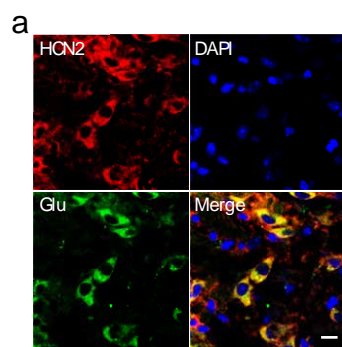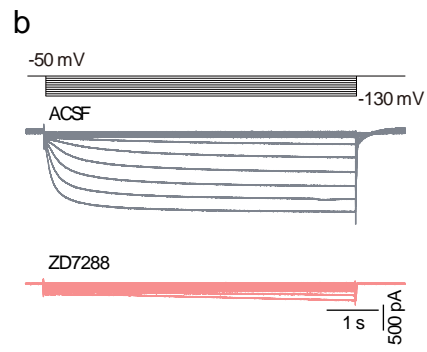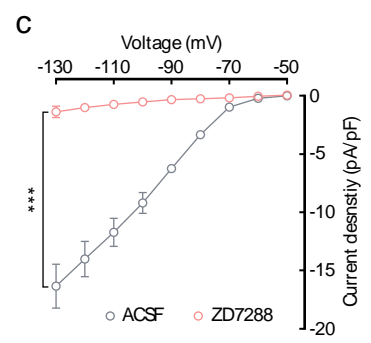

Supplement: nwac275_Supplemental_Files [file nwac275_supplemental_files.zip › Fig. S8.pdf]

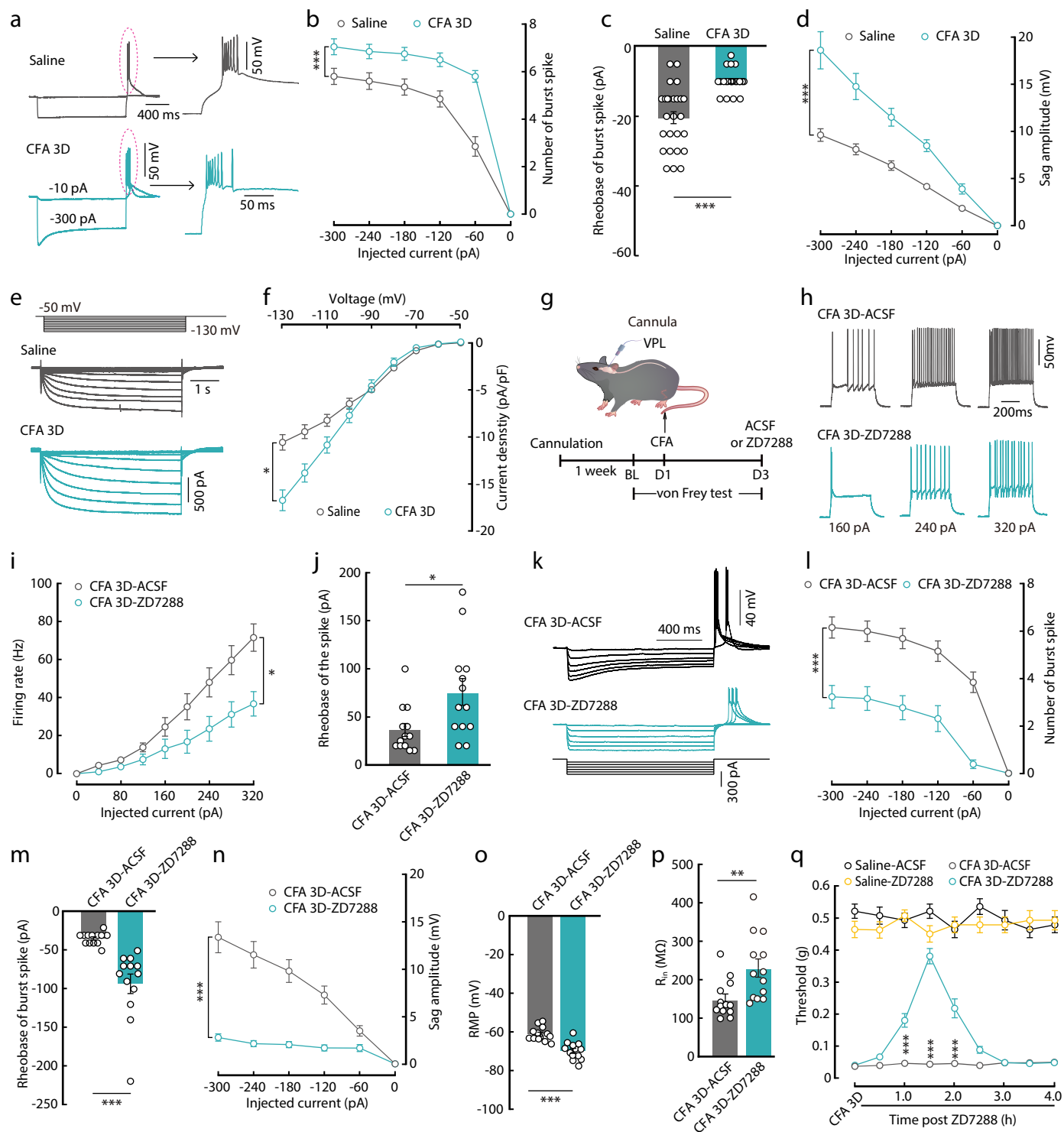

Supplement: nwac275_Supplemental_Files [file nwac275_supplemental_files.zip › Fig. S9.pdf]
